# Two-Dimensional Anisotropic Flexibility of Mechanically Responsive Crystalline Cadmium(II) Coordination Polymers

**DOI:** 10.1021/acs.chemmater.2c00062

**Published:** 2022-02-18

**Authors:** Mateja Pisačić, Ivan Kodrin, Amanda Trninić, Marijana Đaković

**Affiliations:** Department of Chemistry, Faculty of Science, University of Zagreb, Horvatovac 102a, 10 000 Zagreb, Croatia

## Abstract

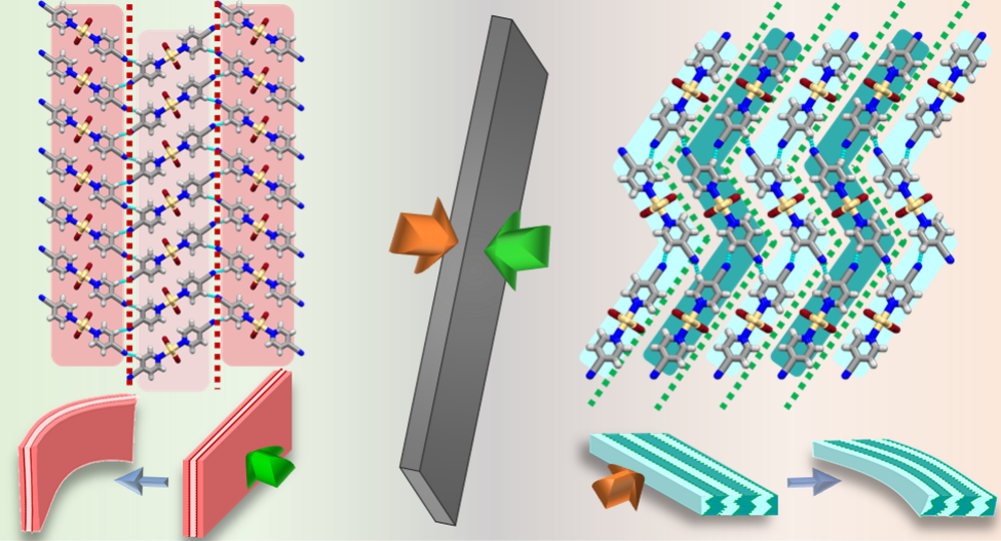

Crystals of a family of six one-dimensional (1D) coordination polymers of cadmium(II) with cyanopyridines [[CdX_2_L_2_]_*n*_, where X = Cl, Br, or I and L = 3-cyanopyridine (3-CNpy) or 4-cyanopyridine (4-CNpy)] presented a variety of morphologies and mechanical responses with dominant two-dimensional (2D) anisotropic flexibility, which has not been previously reported. All mechanically adaptable crystals were 2D flexible and displayed a variety of direction-dependent responses; in addition to 2D isotropic flexibility observed for solely elastic materials, 2D anisotropic flexibility was noticed for both elastic and elastic → plastic crystals. The consequences of fine and controlled structural variations on mechanical behavior were additionally explored via microfocus single-crystal X-ray diffraction and complementary theoretical studies, revealing that the relative strength and direction of the hydrogen bonding interactions were the key parameters in delivering a specific mechanical response.

## Introduction

Brittleness, a property typically associated with molecular crystalline solids, has long hampered the application of these highly ordered solid-state materials in advanced technologies.^1^ Relatively recently, it has been demonstrated that crystalline molecular materials, although quite fragile, under certain circumstances may adapt to a variety of external stimuli (i.e., heat,^2−4^ irradiation,^5−7^ or pressure^8−15^) while maintaining their integrity, which subsequently categorized them as exceptional candidates for application in emerging technologies (i.e., optical waveguides,^16^ electrical conductors,^17,18^ and magnetic devices^19^) and introduced the exploration of crystal flexibility to the forefront of solid-state research.

Coordination polymers (CPs), in particular one-dimensional (1D) CPs, emerged as ideal model systems for exploring structural background and underlying principles that lead to flexible responsiveness and control of a targeted mechanical output of crystalline molecular solids. In the first report on the flexibility of 1D crystalline coordination polymers, we have shown that this particular class of materials is capable of displaying exceptional elasticity in response to an applied external mechanical pressure and that the extent of elastic responses could differ within a family of almost identical substances.^20^ Moreover, the results showed that the extent of the elastic response may correlate with the importance of intermolecular interactions orthogonal to the direction of spreading of 1D polymeric chains. Furthermore, another family of closely related 1D CPs demonstrated that supramolecular interactions also have a substantial impact on plastic deformability and that they may be critical for delivering a spectrum of variable flexible responses.^21,22^

In addition, two types of crystal flexibility have also been observed, 1D and two-dimensional (2D), and these were reported for both elastically and plastically bendable crystals.^10,20−22^ For 1D flexibility, the crystals were easily bent over only one set of prominent crystal faces (viz. bending faces), and they readily broke when the force was applied to the other set of crystal faces. On the contrary, for 2D flexibility, crystals were equally easy bent over both sets of bending faces, thus displaying isotropic flexible responses. This observation urged us to explore the ability of crystals to display 2D anisotropic flexibility, i.e., being 2D flexible but presenting different extents of responses in two bending directions, as such a property might improve their application potential and performance.

With this in mind, we opted for a family of cadmium(II) coordination polymers with pyridine ligands bearing the cyano functionality. We selected two cyano derivatives, 3-cyanopyridine (3-CNpy) and 4-cyanopiridine (4-CNpy), so that we would have a sufficiently diverse set of 1D CPs to examine a potential 2D anisotropy of mechanical responses, and the correlation of those with structural and energy features.

## Results and Discussion

### Structural Characterization

**Figure 1:**
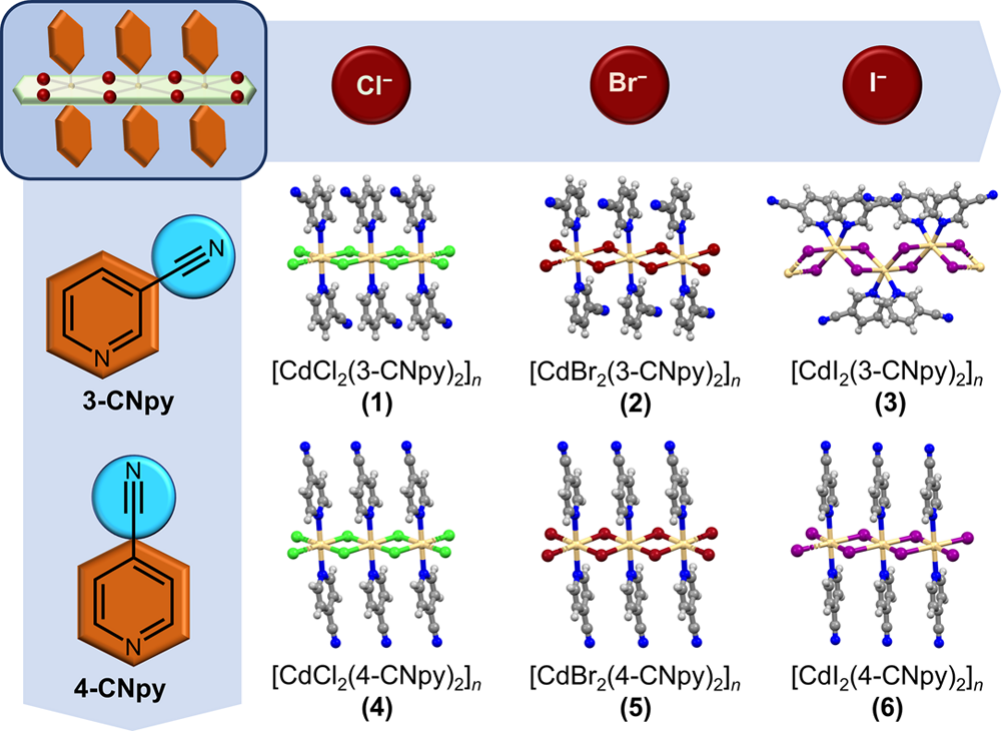
Starting ligands [3-cyanopyridine (3-CNpy) and 4-cyanopyridine (4-CNpy)] and produced 1D coordination polymers listed according to the halide anion used for building up the 1D backbone.

By combining three cadmium(II) halides (CdX_2_, where X = Cl, Br, or I) with two cyano derivatives of pyridine (3-CNpy and 4-CNpy), we were able to deliver crystals of all six CPs with the required morphology and needed quality for testing mechanical behavior, namely, [CdCl_2_(3-CNpy)_2_]_*n*_ (**1**), [CdBr_2_(3-CNpy)_2_]_*n*_ (**2**), [CdI_2_(3-CNpy)_2_]_*n*_ (**3**), [CdCl_2_(4-CNpy)_2_]_*n*_ (**4**), [CdBr_2_(4-CNpy)_2_]_*n*_ (**5**), and [CdI_2_(4-CNpy)_2_]_*n*_ (**6**) (Figure 1).

Structure determination revealed that all materials crystallize in the monoclinic crystal system, the chlorides (**1** and **4**) and bromides (**2** and **5**) in the *P*2_1_/*c* space group and the iodide analogues in the *I*2/*a* (**3**) and *C*2/*m* (**6**) space groups. The materials (**1–6**) consist of the intended 1D building units, having cadmium(II) centers doubly bridged by the halide anions, and propagating in the direction of the “short” crystallographic axis (the *a* axis for **1–5** and *b* axis for **6**). The octahedral geometry around the Cd(II) cations is completed by two cyanopyridine ligands bonded in the *trans* orientation in **1**, **2**, and **4–6** and the *cis* orientation in **3** (Figure 1).

**Figure 2:**
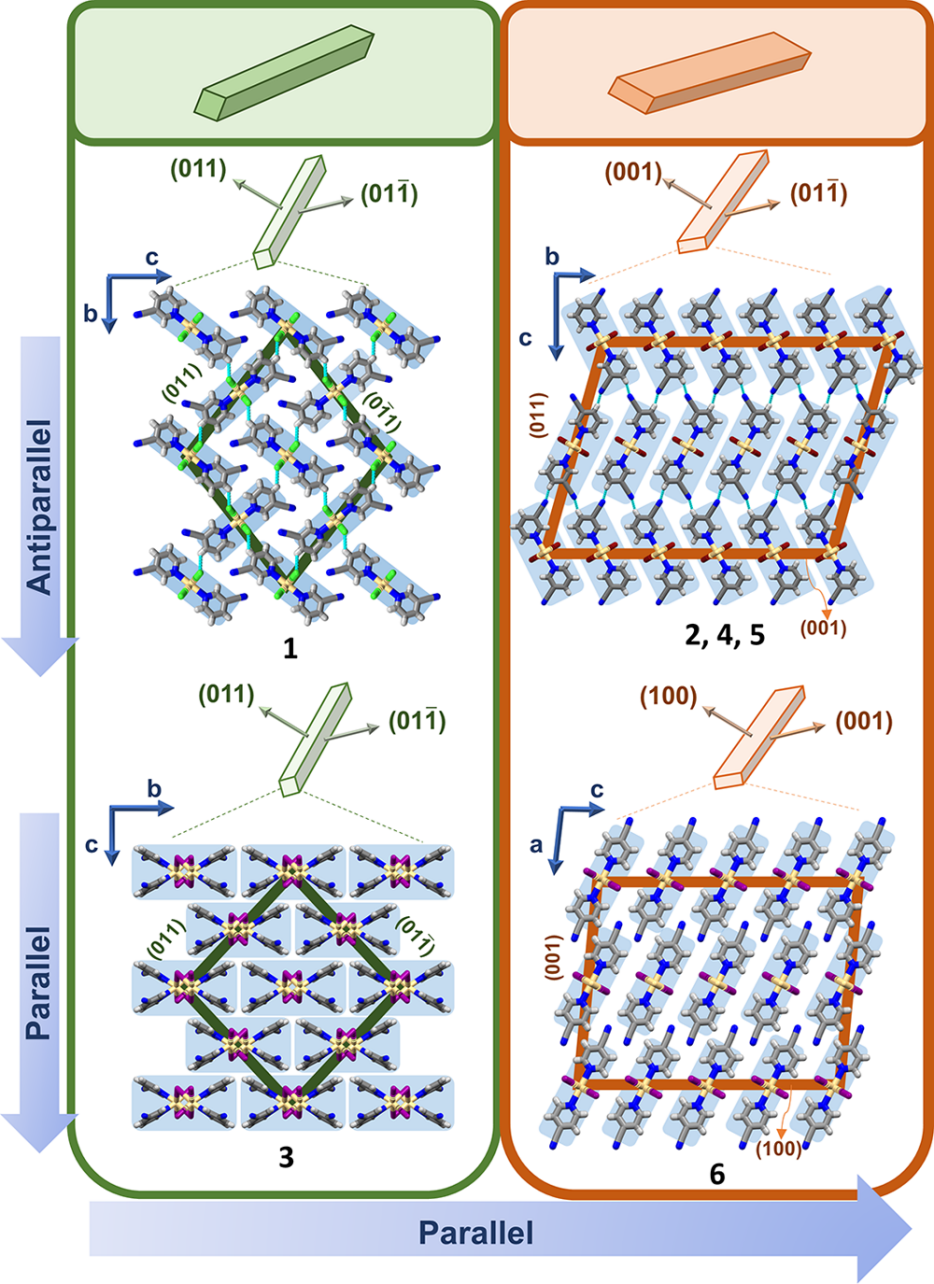
Morphologies and crystal packings of **1–6**. Two equally developed pairs of prominent crystal faces were observed for **1** and **3** (left column), while differently developed ones were found for **2** and **4–6** (right column). Green (**1** and **3**) and red (**2** and **4–6**) thick lines indicate crystal faces that run along the direction of the elongation of the crystal itself. Crystal structures of **2**, **4**, and **5** (right column, top) are nearly identical.

A comparison of the relative orientation of 1D building units in the crystal packing of **1–6** revealed four different types of arrangements (Figure 2). In **1**, 1D chains are arranged in a parallel fashion along the crystallographic *c* axis and antiparallel along the *b* axis, which is like the arrangement observed earlier for two other families of flexible coordination polymers.^20−22^ The C–H···N hydrogen bond was found to be the only dominant intermolecular interaction (with the normalized distance *R*_HX_ < 1);^23^ it links the neighboring 1D chains from antiparallel layers and runs parallel to potential bending faces (indicated by the green lines), i.e., crystal faces (011)/(011)/(011)/(011) (Figure 2, left column, top).

A similar arrangement of the 1D chains was found for three structurally almost identical materials, **2**, **4**, and **5**, with an obvious difference to **1** being displayed in the tilting angle between the antiparallel layers. Here, in addition to the dominant C–H···N interactions, which link the neighboring 1D chains from antiparallel layers, bifurcated C–H···X(Cd) interactions connecting the neighboring polymeric chains within the parallel 2D layers are also present (Figure 2, right column, top; Figure S2). The two interactions run parallel to the directions indicated by the orange lines representing potential bending faces, i.e., crystal faces (001)/(001)/(011)/(011). Both interactions are slightly more influential in **4** than in **2** and **5** (**2**, 0.97 < *R*_HX_ < 1.05; **4**, 0.94 < *R*_HX_ < 1.04; **5**, 0.95 < *R*_HX_ < 1.04).

The 1D building units of the iodide analogues, **3** and **6**, on the contrary, arrange in a parallel manner along both crystallographic axes (**3**, *b* and *c*; **6**, *a* and *c*) perpendicular to the direction of the crystal elongation, i.e., short crystallographic axis (**3**, *a*; **6**, *b*). In **3**, the 1D chains are mutually linked via the C–H···N interactions in the *b* direction, while in **6**, the only noteworthy interactions in the crystal structure are polar interactions between the cyano groups.

### Mechanical Behavior

**Figure 3:**
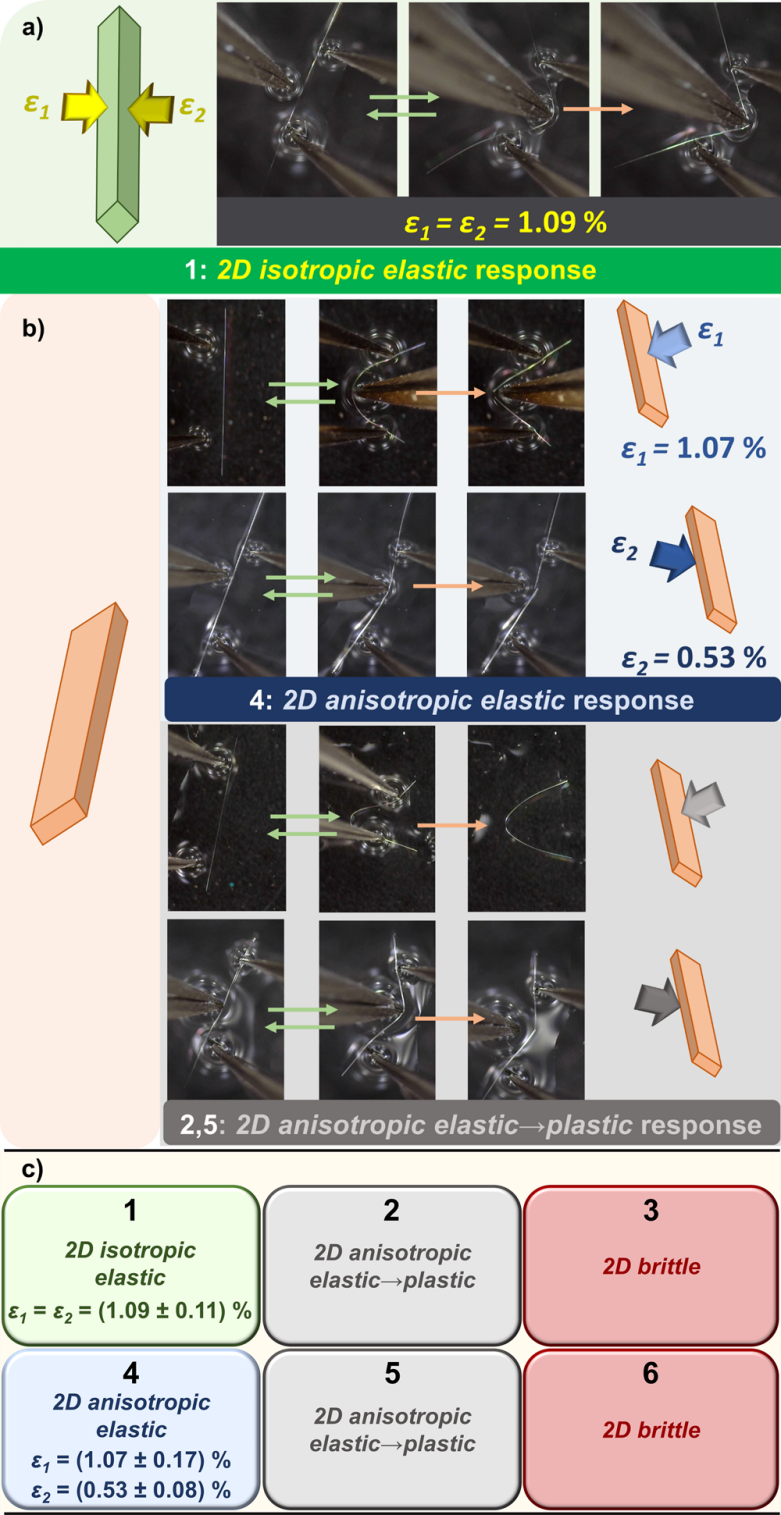
Difference in the observed mechanical response. (a) 2D isotropic elastic response of **1**. (b) 2D anisotropic elastic response of **4** (light blue background) with different extents of bending in response to the application of force to faces of larger dimensions (top row; *ε*_1_ = 1.07%) and smaller dimensions (bottom row; *ε*_2_ = 0.53%). 2D anisotropic elastic → plastic response (light gray background) of **2** and **5** displaying larger deformation when the stress is applied to faces of larger dimensions (top row) than on faces of smaller dimensions (bottom row). (c) Summary of the observed mechanical responses for crystals of **1–6** together with the bending strain values for elastically bendable crystals.

In addition to the diversity of arrangements of 1D building units in **1–6**, a diversity of morphologies of their acicular crystals were also noticed. While crystals of **1** and **3** displayed equally developed prominent crystal faces, i.e., (011)/(011)/(011)/(011) (Figures S17 and S21), a difference in the dimensions of those of **2** and **4**–**6**, (001)/(001) versus (011)/(011), was clearly visible (Figures S19, S23, S26, and S28 and Figure 2). Moreover, a variety of mechanical responses to external force were also observed for the six substances when their crystals were subjected to testing via a modified three-point bending procedure (Figure 3; for more details, see the Supporting Information).

Crystals of **1** displayed typical elastic behavior. Upon application of the mechanical force, crystals bent and regained their original shape once the force was released (Movie 1). The bending–unbending cycles could be repeated a number of times, but when the maximal curvature was exceeded, the crystals broke (Figure S18). Moreover, no perceptible difference in mechanical output was observed when the force was applied to each of the two pairs of bending faces. This places crystals of **1** in the category of 2D isotropically elastic crystals. The quantification of elastic responses (for details, see the Supporting Information), using the Euler–Bernoulli equation, revealed a substantially large bending strain value (ε = 1.09%), confirming the highly elastic flexibility of **1** (Figure S18 and Table S7). Crystals of **3**, on the contrary, were not capable of tolerating external mechanical force in either of the two directions. They immediately broke, thus being classified as 2D brittle (Movie 4 and Figure S22).

As opposed to **1** and **3**, crystals of **2** and **4–6**, due to their differently developed pairs of potential bending faces, could be best described as very elongated plate-like crystals. Therefore, the responsiveness from both pairs was thoroughly examined and the mechanical output was carefully mapped out. Of four compounds in this group, three displayed nearly the same structure (**2**, **4**, and **5**), and we first focused on those three.

Crystals of **4** showed purely elastic responses when bent in both directions but with different extents of elasticity. The crystals were bent to a larger extent when the force was applied to the pair of larger faces [i.e., bending over the thinner bending faces, (001)/(001) (Movie 5 and Figure S24)]. On the contrary, when the force was applied to the smaller crystal faces (i.e., bending over the thicker bending faces), (011)/(011), crystals could tolerate substantially smaller stresses while still displaying an elastic response (Movie 6 and Figure S25). The visual observations were further confirmed by quantification, and two clearly different bending strain values were obtained: *ε*_1_ = 1.07% and *ε*_2_ = 0.53% for bending over (011)/(011) and (001)/(001) pairs of crystal faces, respectively (Table S8). Because crystals of **4** can be bent in two orthogonal directions with a substantial difference in the bending strain values (Δε ≈ 0.5%), they were classified as 2D anisotropically elastic. Moreover, in contrast to previously reported elastic crystals with a clear distinction between the elastic terms, i.e., 1D elastic (being elastically flexible over only one set of bending faces) and 2D elastic [being elastic over both sets of bending faces but with no perceptible difference in bending extent (Δε ≈ 0%)], here we introduce (an)isotropicity in the 2D term to stress the difference between those two types of 2D elastic behavior, namely, 2D anisotropic elasticity and 2D isotropic elasticity.

In contrast, crystals of the bromide analogues (**2** and **5**), although having crystal packing almost identical to that of **4**, presented somewhat different behavior. Upon exposure, crystals of both **2** and **5** readily adapted to mechanical force resembling the behavior of **4**, but upon removal of the force, they could restore their original shape only partially, thus displaying a slight plastic deformation. Surprisingly, similar behavior was observed when the force was applied to both larger, (001)/(001), and smaller, (011)/011), bending faces but with a clear anisotropy of the extent of bending. Here as well, crystals could be bent substantially more if bent over the thinner [i.e., force exerted on the larger face, (001)/(001)] then over the thicker face.

Moreover, when bent over both thicker and thinner crystal faces and achieving only smaller curvatures, crystals could restore their unbent shape completely. However, with further application of the mechanical stress, crystals remain permanently deformed, and if bent even more, they finally broke (**2**, Movie 2 and Figure S20; **5**, Movie 7 and Figure S27). When all of the observations are taken into account, it is clear that crystals of **2** and **5** can be categorized as 2D anisotropically elastic → plastic.

Finally, crystals of **6**, like those of **3**, showed brittle behavior when the force was applied to both pairs of prominent crystal faces (Movies 9 and 10 and Figure S29); thus, crystals of **6** were also 2D brittle.

### Structure–Mechanical Property Correlation

Of the six examined materials, here we first focused on the three with almost identical crystal packing (**2**, **4**, and **5**) as they enabled a correlation of fine structural details with mechanical behavior. Moreover, the three materials displayed a newly observed directional dependence of crystal adaptability on mechanical stimuli, i.e., 2D anisotropy of mechanical behavior. This was most clearly demonstrated for **4** where the difference in the extent of elasticity was confirmed by quantification (ε_1_ = 1.07%, and ε_2_ = 0.53%) by which the impact of crystal dimensions was eliminated, and what in turn clearly showed that the difference in bending was primarily related to the anisotropy of the structural features in the crystal structure.

**Figure 4:**
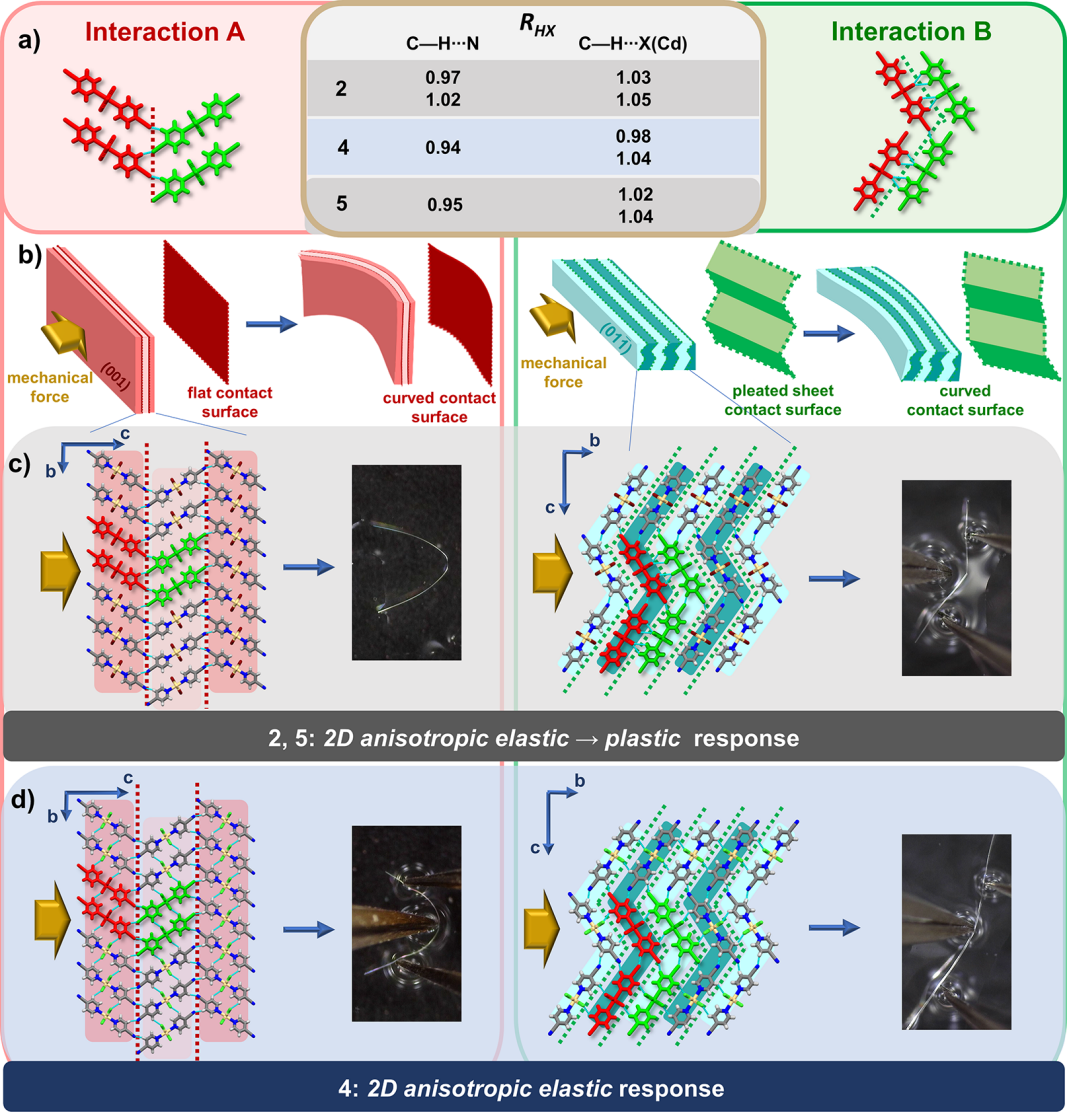
(a) Double pairs of 1D polymeric chains, with regions of weak interactions indicated as interaction A (red dotted lines) and interaction B (green dotted lines). The intermolecular interactions shorter than the sum of van der Waals radii are shown as cyan dotted lines and listed in the table with normalized distance *R*_HX_ for **2**, **4** and **5**. (b) Schematic presentation of direction dependent bending process and slicing the crystal up into 2D molecular layers parallel to the larger face (dark/pale red layers), with 2D regions of weak interacions presented as flat red contact surfaces (left), and parallel to the smaller face (dark/pale blue, zigzag layers), with 2D regions of weak interactions presented as green corrugated contact surfaces (right). (c) Crystal structure of **5** (c) and **4** (d) sliced up into 2D molecular layers parallel to crystal faces of larger dimensions face(highlighted withdark/pale red background, left) and **smaller dimensions** (zigzag layers, highlighted with dark/pale green background, right). Next to the highlighted molecular layers for each compound, the mechanical output of the application of the mechanical force to the respective pair of faces is presented.

To shed more light on the origin of the observed anisotropy of mechanical output, we focused on the fine details in their supramolecular architectures, in particular the details in the regions of weaker interactions parallel to the bending faces. As crystals presented two sets of differently developed bending faces, two regions of weak interactions were identified and indicated by the red and green dotted lines (Figure 4) and termed as interactions **A** (forming flat 2D planes) and interactions **B** (forming corrugated 2D regions), respectively.

In **2**, **4**, and **5**, the same types of intermolecular interactions, namely, C–H···N and bifurcated C–H···X(Cd), materialized but they were of different relative importance. While in the regions parallel to the larger crystal faces only C–H···N interactions were observed (**A**, red dotted lines), in the regions parallel to the smaller crystal faces (**B**, green dotted lines) both types were present, making **B** regions somewhat stronger, which in turn, together with the corrugation of **B**, caused crystals to be less tolerable to bending over the (011)/(011) faces.

In addition, two types of 2D anisotropic mechanical responses were also observed within the group of the three materials (**2**, **4**, and **5**), 2D anisotropic elastic response (**4**), and 2D anisotropic elastic → plastic response (**2** and **5**). If we further focus on the relative importance of each of the two interactions among the three materials, it is easy to notice that in **2** and **5** both interactions are less influential (being longer and less linear) than in **4**, which makes layers in **2** and **5** more prone to slip over each other once the critical radius is exceeded, thus displaying elastic → plastic behavior in contrast to solely elastic behavior of **4**. This is in line with our previous findings where for the family of isostructural compounds the strength of interactions accounted for the variations in mechanical output, and where the weaker interactions allowed the slippage of neighboring slabs over each other.^21,22^

On the contrary, crystals of **1**, while, like **4**, being also pronouncedly elastic, were the only 2D isotropically responsive. Although arranged in antiparallel 2D layers as in **4**, 1D building units from neighboring layers in **1** were more tilted to each other, which in turn resulted in equally developed crystal faces and an almost identical arrangement of molecular and intermolecular features parallel to bending faces, as well as in the directions of the application of force.

Finally, crystals of **3** and **6** were both 2D brittle. Unfortunately, due to the lack of materials with identical and/or similar crystal packing (as of **3** and **6**) within the examined set of compounds, we were not in a position to rationalize their behavior and draw any conclusion.

### Rationalization of Mechanical Behavior against the Backdrop of Calculated Energies

To deepen our understanding of the relationship between macroscopic responses and microscopic features, we focused solely on three materials with almost identical crystal packing, **2**, **4**, and **5**. First, we opted to examine the strength of intermolecular interactions in two 2D regions (spreading parallel to the bending faces) indicated by red and green dotted lines (Figure 4), as these are the weakest regions in the crystal structure, and as such, they are the first barriers in preserving the crystal integrity upon its exposure to external pressure. Therefore, we calculated basis set superposition error-corrected (BSSE) interaction energies between the double pairs of adjacent molecular fragments [red and green double pairs (Figure 4); for details see Figures S30 and S31].

The interactions B (−122.7 to −160.0 kJ/mol) proved to be much stronger than the interactions A (−49.7 to 57.5 kJ/mol), as a consequence of a substantially larger contact area as well as a larger number of HBs in B than in A [three C–H···N bonds in A vs one C–H···N bond and eight C–H···X(Cd) bonds in B]. This in turn made the bending much more restrictive when the force was applied to the smaller crystal face than to the larger one.

Furthermore, interaction energy B for the bromide analogues (**2**, −122.7 kJ/mol; **5**, −143.7 kJ/mol) was noticeably smaller than for the chloride one (**4**, −160.0 kJ/mol), which made the weak regions in **2** and **5** weaker than in **4**, and consequently, the adjacent domains more prone to slips over each other, thus making **2** and **5** elastic → plastic and **4** being only elastic. On the contrary, the energies of A do not offer equally clear rationalization due to the distribution of energies being within a small energy range (−50 to −58 kJ mol^–1^), but rather suggesting that the concerted fashion of the interactions in both regions is most likely to be needed for the crystal to display a flexible response.

### Microfocus Synchrotron X-ray Experiments

**Figure 5:**
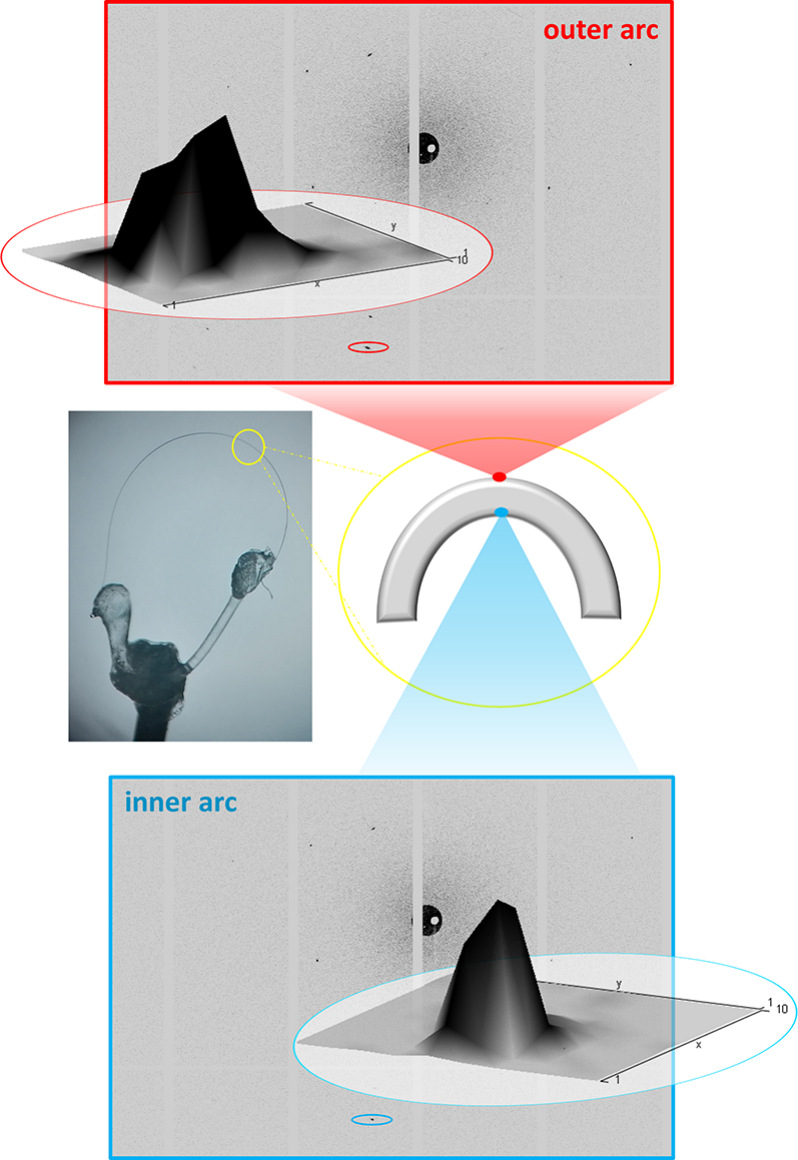
Profiles of the diffraction peaks of **1** (middle left) and the outer (top) and inner (bottom) arcs of the bent crystal.

The crystal of **1**, the only material with 2D isotropically elastic crystals, was examined by microfocus synchrotron X-ray radiation with the aim of gaining insight into the molecular-level consequences of the bending process. The crystal was mounted on a glass holder in its bent form, oriented in the way that the X-ray beam was orthogonal to the plane of the loop of the crystal, and data were collected at two points of the bent crystal, the inner and outer arc, at the region of its maximal curvature (Figure 5).

The unit cell parameters were determined at the two points and compared with those from the straight crystal (prior bending), which confirmed that the crystal integrity was preserved, and no phase changes were introduced by bending.^24^ The data revealed a noticeable enlargement of the *a* axis at the outer arc and its contraction at the inner arc (5σ), while changes in the other unit cell parameters upon bending were less reliable (<3σ). The changes in the *a* axis, in turn, indicated elongation of the Cd(II)···Cd(II) distances within the 1D polymeric chains at the outer part and shortening at the inner part of the crystal. Similar shortening and elongation at the inner and outer arcs of the bent crystal, respectively, were observed for elastically bendable crystals composed of discrete zero-dimensional (0D) metal-based building units.^11^ However, in contrast to our materials, the distance between the individual molecules in the 0D material was found to remain constant, while molecules rotate in the opposite directions, with respect to each other, at the two arcs of the crystal to compensate for extension and compression of the crystal parts (convex and concave) during bending. Although our findings suggest a different mechanism of elastic flexure for 1D materials (in comparison with the 0D material), they further point to the importance of concerted action of intermolecular interactions spreading in the two orthogonal directions (i.e., in regions A and B) for the crystal to display a flexible response and preserve the crystal integrity. While microfocus synchrotron experiments shed light on the molecular-level consequences of elastically bent crystals, for plastically bent crystals, where structure perturbations occur during the bending process, an alternative approach would be required, i.e., microfocus infrared spectroscopy with synchrotron radiation coupled with density functional theory (DFT) computation.^25^

Another interesting feature was also noticed as a consequence of bending, and it became visible upon examination of the peak profiles from two opposite parts (inner and outer part) of the bent crystal. While peaks from both parts were broadened (in comparison with the unbent crystal), broadening from the outer arc was much more pronounced than that from the inner arc (Figure 5). That in turn suggests that the bending process introduced larger distortions at the outer than at the inner region of the bent crystal. To further examine the observation, we opted for calculation of energies that would accompany distortions of the unit cells.

### Potential Energy Surface and Their Relationship with the Unit Cell Distortions

To calculate the energy profiles that would accompany the distortions of the unit cells, we again opted for **2**, **4**, and **5** as they enabled the correlation of the results with structural features and experimental findings.

**Figure 6:**
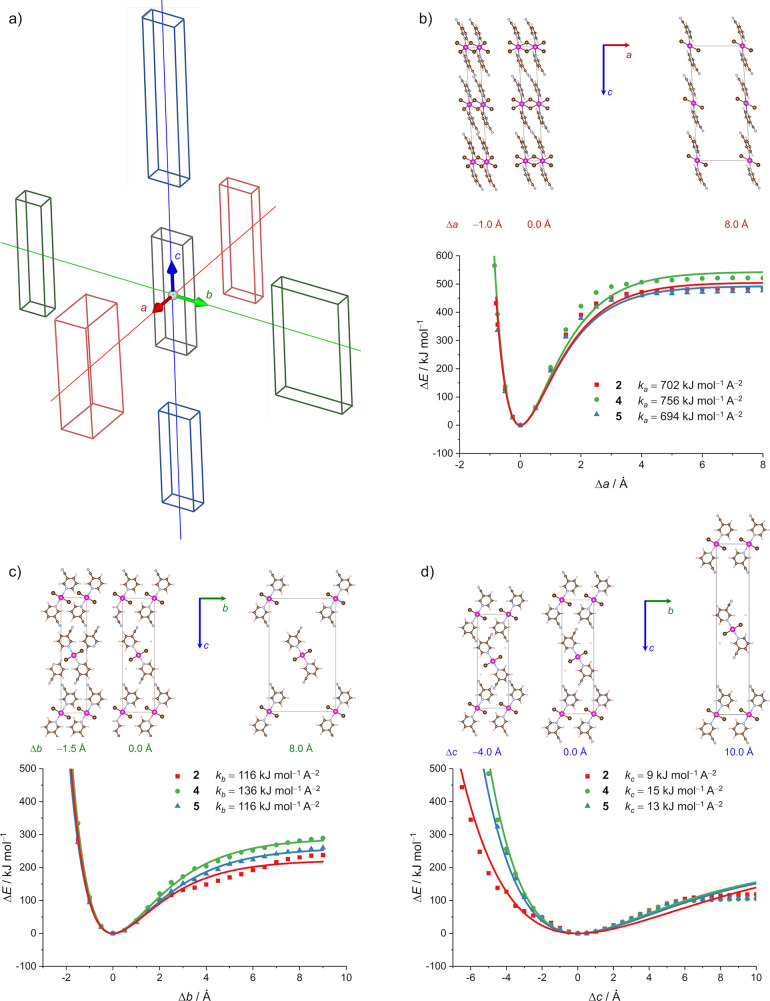
(a) Deformation of the unit cells by monodirectional stretching and shrinking along the unit cell axis. (b–d) Potential energy curves for **2** (red), **4** (green), and **5** (blue) as a function of relative deformation of the unit cell along the *a*, *b*, and *c* axes. The zero values of Δ*a*, Δ*b*, and Δ*c* correspond to the fully optimized geometries at the PBE-D3/pob-TZVP-rev2 level of theory. The solid lines represent fits to a conventional Morse potential from which force constants *k*_*a*_, *k*_*b*_, and *k*_*c*_ were calculated.

Each unit cell parameter was changed in regular increments in a separate experiment, and the three energy profiles were retrieved referring to the relative deformation of the unit cell lengths (*a*, *b*, or *c*) describing the process of extending and shrinking the unit cell axis [relative to the optimized structure corresponding to the minimum on the potential energy surface (Figure 6a); for details, see Computational Studies]. The energy profiles were then fitted to the Morse potential, which allowed us to calculate the force constants, *k*_*a*_, *k*_*b*_, and *k*_*c*_ [along the *a*, *b*, and *c* axes, respectively (Figure 6b–d)], and to directly compare those for the three materials (**2**, **4**, and **5**) as well as to correlate them with the strength of monodirectional deformation of the unit cell along each axis. For all three materials, the calculated energy profiles indeed nicely resembled the Morse potential, which in turn also supported the smaller distortion to be more likely to materialize at the contraction part and larger distortions at the extension part of the crystal (when somewhere larger unit cell distortions materialize).

Moreover, the largest *k* values (**2**, **4**, and **5**) were, unsurprisingly, derived for the deformations along the shortest axis (*a* axis; *k*_*a*_) coinciding with the direction of spreading 1D polymeric chains. A slightly larger *k*_*a*_ value for **4**, in comparison with those of **2** and **5** (**2**, 702 kJ mol^–1^ A^–2^; **4**, 756 kJ mol^–1^ A^–2^; **5**, 694 kJ mol^–1^ A^–2^), reflected that it is more difficult to achieve the deformation in the direction of the *a* axis for the chloride (**4**) than for the bromide derivatives (**2** and **5**), which is in line with the stronger Cd–X bonds in **4** (X = Cl) than in **2** and **5** (X = Br).

On the contrary, the force constants for the deformation processes along the other two directions, the *b* and *c* axes, are notably smaller due to the intermolecular interactions being the sole factors for determining the connectivity strength in those directions. The force constants *k*_*b*_ were larger than *k*_*c*_ for all three materials (*k*_*b*_ ≈ 120 kJ mol^–1^ A^–2^; *k*_*c*_ ≈ 12 kJ mol^–1^ A^–2^), pointing at the deformations along the *b* axis to be more difficult to achieve than the ones along the *c* axis. Indeed, the bending strain in **4** was 1.07% (*ε*_1_) for applying the force to the (001)/(001) pair of crystal faces, which fitted quite nicely with the lower value of the calculated *k*_*c*_ of 15 kJ mol^–1^ A^–2^. The bending strain was halved (*ε*_2_ = 0.53%) when the force was applied to the (011/011) face, roughly corresponding to the larger *k*_*b*_ value of 136 kJ mol^–1^ A^–2^ (as in that case the component promoting the deformation along the *c* direction also contributed, not only the one along the *b* direction).

Furthermore, somewhat larger values for both constants (*k*_*b*_ and *k*_*c*_) were found for **4** than for **2** and **5**, thus making **4** more prone to resist slipping of adjacent layers over each other than is the case for **2** and **5**. This finding is again quite in line with our experimental observations, where **4** was solely elastic while **2** and **5** showed elastic → plastic responsiveness.

## Conclusion

The family of six coordination polymers of cadmium(II) equipped with the cyanopyridine ligands provided us with a diverse data set of crystal morphologies and crystal responses that in turn yielded a newly described anisotropy in 2D flexibility of crystals. This finding prompted us to elucidate the origin of this surprising crystalline property and to rationalize it against a variety of structural, morphological, and energy features.

The six materials displayed four different arrangements of 1D building units with two different morphologies. Of six, four materials presented flexible responses, while two were brittle. All four flexible materials were 2D responsive, and their responsiveness showed a correlation with crystal morphologies; the crystals with equally developed crystal faces were 2D isotropically flexible, while materials with elongated plate-like crystals, for the first time, yielded 2D anisotropically flexible crystals, i.e., displayed a direction-dependent crystal adaptability to mechanical stimuli. More interestingly, among the three anisotropically responsive materials, a variety of responses were also observed, one material being solely elastic, while two displayed a transition from elastic to plastic behavior (elastic → plastic) at larger curvatures. The intermolecular interactions, together with structural and energy features, proved to be instrumental in delivering this assortment of crystal adaptabilities to mechanical stress. Small variations in the tilting angle between the 1D polymers from neighboring layers guided the crystal morphology, needle-like versus elongated plate-like crystal morphology, which, together with the intermolecular interactions and the energy thereof, determined the mechanical output. For a given material, the increase in the interaction energy in orthogonal directions accompanied by the corrugated arrangement of the building units proved to be critical for the weakened crystal ability to bend, which resulted in the 2D anisotropic flexibility of the crystal. On the contrary, for different materials (within the almost identical group of substances), the increase in the interaction energies (in identical directions) was followed by an improved ability to resist plastic deformation.

This newly described 2D anisotropic mechanical responsiveness of crystals together with the findings on the origin thereof will advance the engineering and delivery of targeted mechanical responses, thus making the crystalline materials at disposal for practical applications in advanced technologies.

## Experimental Section

### Crystallization Experiments

Crystals of compounds **1–6** were prepared by the layering technique. Cadmium(II) salt (CdX_2_, 1 equiv) was dissolved in water, added to a test tube, and carefully layered with 1 mL of pure ethanol and then with an ethanol solution of the ligand (3-CNpy or 4-CNpy, 2 equiv). In a few weeks, needle-like crystals were obtained.

### PXRD

X-ray powder diffraction experiments were performed on a Malvern Panalytical Aeris powder diffractometer under an applied voltage of 40 kV and a current of 15 mA, with Cu Kα radiation. The patterns were collected in the angle region between 5° and 50° (2θ) with a step size of 0.02°.

### SCXRD

Crystals of **1–6** were mounted on a glass fiber and glued with superglue. Data were collected at room temperature, 295(2) K, on an XtaLAB Synergy-S Dualflex diffractometer equipped with a PhotonJet (Mo) microfocus X-ray source and a HyPix-6000HE hybrid photon counting (HPC) X-ray area detector. Data collection and reduction, including absorption correction, were performed using CrysAlisPro.^26^ The structures were determined using the Olex2 interface. The starting structural model was obtained using SHELXT^27^ and refined with the SHELXL algorithm.^28^

### Synchrotron Measurements: Mapping Out Slight Structural Changes

Data were collected on the XRD1 beamline at synchrotron Elettra (Trieste, Italy).^29^ All measurements were performed at room temperature using a wavelength λ of 0.7000 Å. A 120 μm × 100 μm X-ray beam was prealigned and masked with a pinhole being brought down to a size of approximately 5 μm in diameter (full width at half-maximum). The crystal was glued in a bent form on a magnetic base holder and placed on a goniometer head. The crystal was oriented in a way that the trajectory of the beam was perpendicular to the loop of the crystal. A point at the maximal curvature of the bent crystal was selected, and the crystal was positioned in two ways so that only a small portion of the outer and inner arc of the crystal was in the beam. The unit cell parameters were determined in those two positions of the bent crystal (at the outer and inner arc of a bent crystal) by collecting 12 diffraction frames with an oscillation angle of 0.5° (total of 6°) and an exposure time of 30 s. Data reductions were performed using CrysAlisPro.^26^ For the outer arc, *a* = 3.811(5) Å, *b* = 15.53(14) Å, *c* = 11.60(3) Å, α = 90°, β = 91.51(8)°, γ = 90°, and *V* = 687(6) Å^3^. For the inner arc, *a* = 3.770(7) Å, *b* = 15.62(15) Å, *c* = 11.59(3) Å, α = 90°, β = 91.37(10)°, γ = 90°, and *V* = 682(7) Å^3^.

### TG/DSC

Thermal analyses were performed using a simultaneous TGA-DTA analyzer (Mettler-Toledo TGA/DSC 3+). Finely ground samples (**1–6**) were placed in alumina pans (70 μL) and heated in flowing nitrogen (50 mL min^–1^) from room temperature to 600 °C at a rate of 10 °C min^–1^. Data collection and analyses were performed using the program package STARe Software 15.01 (MettlerToledo GmbH, 2015).^30^

### Mechanical Adaptability Testing

Tests of mechanical responses of prepared crystals were performed via the modified three-point bending procedure. Several crystals of each compound, from a few different batches, were selected. Each selected crystal was placed on a glass slide and immersed in a small amount of paratone oil to reduce the damage of the crystal upon the usage of metalware and to avoid crystal–surface friction. The crystal was held with a pair of metal forceps from one side, while the mechanical force was applied from the opposite side, using a metal needle. The force was applied until the crystal broke. For crystals that displayed an elastic response, the extent of the response was quantified using the Euler–Bernoulli equation.

### Computational Studies

Periodic DFT calculations were performed for the crystal structures of coordination polymers **2**, **4**, and **5** in CRYSTAL17^31^ using the PBE^32^ functional with Grimme’s D3 correction for the inclusion of weak dispersive interactions.^33^ The revised triple-ζ basis set specifically adapted for periodic calculations, pob-TZVP-rev2, was used on all atoms.^34^ The input files were generated by the cif2cell package.^35^ Full optimization of atom coordinates and cell parameters was performed on the starting geometries with tighter energy convergence criteria (10^–8^) and root-mean-square values on gradient (6 × 10^–5^) and displacement (1.2 × 10^–4^). Tighter convergence on total energy (10^–7^) and increased truncation criteria for the calculation of Coulombs and exchange integrals (8 8 8 8 16) were set for SCF calculations. For all three compounds, the reciprocal space was sampled using 8 × 4 × 1 Pack–Monkhorst *k*-point mesh (the *c* axis was over 26 Å).

To rationalize the mechanical behavior of **2**, **4**, and **5**, the potential energy surfaces associated with monodirectional deformation (stretching and/or shrinking) along the unit cell axes (*a*, *b*, and *c*) were modeled. We started from fully optimized structures and performed relaxed scan calculations. The starting geometry of each point on the energy profile was created by deformation (stretching or shrinking) of one unit cell parameter (unit cell length *a*, *b*, or *c*) at a time in increments of 0.5 Å (smaller increments were employed around the equilibrium distance), while all other unit cell parameters were kept constant. Interatomic distances were not changed during this process. These starting geometries were then partially optimized (the unit cell parameters were fixed, while atomic positions were allowed to change), and exactly 15 optimization steps were allowed to obtain more realistic energies when compared to values obtained from single-point calculations on nonrelaxed geometries. Calculated energy values were then fitted to the conventional Morse potential function *D*_e_[1 – e^–*a*(*X*–*R*_e_)^], and the force constant was calculated as *k* = 2*D*_e_*a*^2^.

It is worth mentioning that the unit cell is not equally sensitive to the deformations along two directions, namely, the *b* and *c* directions. While the deformation along the *b* axis is in direct relation with the relative displacement of two neighboring polymeric chains within the unit cell (i.e., deformation of the unit cell by 1.0 Å will increase the intrachain distance by the same amount), the deformation along the *c* axis and the separation of the 1D chains differ by a multiplier of 2 (a 1.0 Å deformation of the unit cell along the *c* axis will separate the adjacent chains by only 0.5 Å) due to the presence of two 1D polymers along the *c* axis. Thus, the energy decreases and increases much slower when the unit cell is stretched and shrunk, respectively, by the same amount in direction *c* in comparison with direction *b*, resulting in a substantially smaller force constant value.

Interaction energies were calculated in Gaussian 16^36^ between the selected double pairs (red-green) on fully optimized geometries obtained from periodic DFT calculations as previously described. Each adjacent 1D polymeric chain was modeled as a finite electroneutral molecule of three metal centers, and all of the calculated values were corrected by BSSEs according to the counterpoise method of Boys and Bernardi.^37,38^ The calculated interaction energy values were divided by 3 to obtain the normalized interaction energies per metal center.

Geometries were visualized in GaussView 6^39^ and VESTA.^40^

## Supporting Information Available

Synthetic procedures for the preparation of compounds **1–6**, SCXRD results, PXRD results, TG/DTA analysis, crystal bending experiments, and computational studies (PDF)
Movie 1 (MP4)
Movie 2 (MP4)
Movie 3 (MP4)
Movie 4 (MP4)
Movie 5 (MP4)
Movie 6 (MP4)
Movie 7 (MP4)
Movie 8 (MP4)
Movie 9 (MP4)
Movie 10 (MP4)
