# Correction to Two-Dimensional Anisotropic Flexibility of Mechanically Responsive Crystalline Cadmium(II) Coordination Polymers

**DOI:** 10.1021/acs.chemmater.2c00666

**Published:** 2022-03-23

**Authors:** Mateja Pisačić, Ivan Kodrin, Amanda Trninić, Marijana Đaković

An additional sentence has been added to the Acknowledgments paragraph. The full Acknowledgments paragraph is presented here.

