# Supplementary material for: Two-Dimensional Anisotropic Flexibility of Mechanically Responsive Crystalline Cadmium(II) Coordination Polymers
Source: Chem Mater. 2022 Feb 18;34(5):2439–48. doi: 10.1021/acs.chemmater.2c00062 (PMC8910440; doi:10.1021/acs.chemmater.2c00062)
Supplement: Supplementary file 1 — cm2c00062_si_001.pdf [file cm2c00062_si_001.pdf]

Two-dimensional Anisotropic Flexibility of Mechanically Responsive Crystalline Cadmium(II) Coordination Polymers

Mateja Pisačić,[a] Ivan Kodrin,[a] Amanda Trninić,[a] and Marijana Đaković*[a]

[a]*Department of Chemistry, Faculty of Science, University of Zagreb, 10000 Zagreb, Croatia*

*Corresponding author: mdjakovic@chem.pmf.hr

Supplementary information

Table of Contents

1. Synthesis 3
2. Single crystal X-ray crystallography 5
3. Powder X-ray crystallography 14
4. Thermal analysis (TGA/DTA) 18
5. Testing mechanical responses of prepared crystals 21
6. Computational studies 32
7. References 33

1. Synthesis

Preparation of bulk samples of 1–6.

All reagents and solvents used for synthesis were purchased from commercial suppliers and used without further purification. Cadmium(II) salt (~ 0.5 mmol) was dissolved in 5 mL of water, and mixed with the ethanol solution of the corresponding ligand, 3-cyanopyridine or 4-cyanopyridine (~ 1.0 mmol of the ligand dissolved in 10 mL of ethanol) (Table S1). The mixture was stirred for 30 minutes, after which the resulting white powder was filtered, and washed with a small amount of cold water and ethanol.

The powder diffraction pattern of all samples was consistent with the pattern calculated from the single-crystal data (Figures S3–S8).

Table S1. The amounts of the cadmium(II) salt (dissolved in 5 mL of water) and the ligand (dissolved in 10 mL of ethanol) used for the synthesis of the compounds **1**–**6**, together with the microanalysis results of the products.

Compound	CdX_2 *m* *n*	Ligand *m* *n*	Microanalysis calculated	Microanalysis found
$[CdCl_2(3\text{-}CNpy)_2]_n$ **1**	$CdCl_2·H_2O$ 109 mg 0.54 mmol	**3-CNpy** 106 mg 1.02 mmol	*w*(C) = 36.81% *w*(H) = 2.06% *w*(N) = 7.16%	*w*(C) = 36.85 % *w*(H) = 2.02 % *w*(N) = 7.24 %
$[CdBr_2(3\text{-}CNpy)_2]_n$ **2**	$CdBr_2·4H_2O$ 182 mg 0.53 mmol	**3-CNpy** 110 mg 1.06 mmol	*w*(C) = 29.97 % *w*(H) = 1.68 % *w*(N) = 11.65%	*w*(C) = 29.92 % *w*(H) = 1.70 % *w*(N) = 11.69%
$[CdI_2(3\text{-}CNpy)_2]_n$ **3**	CdI_2 179 mg 0.49 mmol	**3-CNpy** 103 mg 1.00 mmol	*w*(C) = 36.81% *w*(H) = 1.04% *w*(N) = 3.61%	*w*(C) = 36.76% *w*(H) = 1.02% *w*(N) = 3.55%
$[CdCl_2(4\text{-}CNpy)_2]_n$ **4**	$CdCl_2·H_2O$ 103 mg 0.51mmol	**4-CNpy** 112mg 1.08 mmol	*w*(C) = 15.47% *w*(H) = 2.06% *w*(N) = 7.16%	*w*(C) = 15.51% *w*(H) = 2.07% *w*(N) = 7.19%
$[CdBr_2(4\text{-}CNpy)_2]_n$ **5**	$CdBr_2·4H_2O$ 175 mg 0.51 mmol	**4-CNpy** 107 mg 1.03 mmol	*w*(C) = 29.97 % *w*(H) = 1.68 % *w*(N) = 11.65%	*w*(C) = 29.96 % *w*(H) = 1.65 % *w*(N) = 11.63%
$[CdI_2(4\text{-}CNpy)_2]_n$ **6**	CdI_2 190 mg 0.52 mmol	**4-CNpy** 109 mg 1.05 mmol	*w*(C) = 36.81% *w*(H) = 1.04% *w*(N) = 3.61%	*w*(C) = 36.83% *w*(H) = 1.05% *w*(N) = 3.58%

Growing Crystals

General procedure for preparation of single crystals by layering technique

An aqueous solution of cadmium(II) salt (CdX_2, 1 eq.) was added to a test tube and carefully layered with 1 mL of pure ethanol. Lastly, ethanol solution of a ligand (2 eq.) was added to an ethanol layer, and test tubes were sealed with parafilm, and left undisturbed. After a few weeks, colorless needle-like crystals were obtained.

$[CdCl_2(3\text{-CNpy})_2]_n$, (**1**). Used: $CdCl_2$ aqueous solution (1 mL, 0.052 mol dm^{-3}), 3-CNpy ethanol solution (2 mL, 0.051 mol dm^{-3}).

$[CdBr_2(3\text{-CNpy})_2]_n$, (**2**). Used: $CdBr_2$ aqueous solution (1 mL, 0.049 mol dm^{-3}), 3-CNpy ethanol solution (2 mL, 0.050 mol dm^{-3}).

$[CdI_2(3\text{-CNpy})_2]_n$, (**3**). Used: CdI_2 aqueous solution (1 mL, 0.053 mol dm^{-3}), 3-CNpy ethanol solution (2 mL, 0.054 mol dm^{-3}).

$[CdCl_2(4\text{-CNpy})_2]_n$, (**4**). Used: $CdCl_2$ aqueous solution (1 mL, 0.050 mol dm^{-3}), 4-CNpy ethanol solution (2 mL, 0.048 mol dm^{-3}).

$[CdBr_2(4\text{-CNpy})_2]_n$, (**5**). Used: $CdBr_2$ aqueous solution (1 mL, 0.051mol dm^{-3}), 4-CNpy ethanol solution (2 mL, 0.053 mol dm^{-3}).

$[CdI_2(4\text{-CNpy})_2]_n$, (**6**). Used: CdI_2 aqueous solution (1 mL, 0.052 mol dm^{-3}), 4-CNpy ethanol solution (2 mL, 0.049 mol dm^{-3}).

2. Single crystal X-ray crystallography

Crystal structure determination.

Single crystals (**1−6**) were mounted in a random orientation on a glass fiber using superglue. Data collections were carried out at a room temperature (295(2) K) on an XtaLAB Synergy-S Dualflex diffractometer equipped with PhotonJet (Mo, λ = 0.71073 Å) microfocus X-ray source and HyPix-6000HE hybrid photon counting (HPC) X-ray area detector. CrysAlisPro Software was used to control the diffractometer, and to perform data reduction, including absorption correction. The structures were solved in Olex2 software by SHELXT program.[1] The coordinates and the anisotropic thermal parameters for all non-hydrogen atoms were refined by full-matrix least-squares methods based on F^2 using the SHELXL program.[2] The hydrogen atoms were generated geometrically using the riding model with the isotropic factor set at $1.5U_{eq}$.

The visualization of the obtained structural models was done by Mercury 4.3.1.[3] The thermal ellipsoids were drawn at the 50 % probability level. General and crystal data with the summary of intensity data collection and structure refinement for compounds **1−6** are given in Table S2 and Table S3 (ESI).

CCDC 2127796-2127801 contain the supplementary crystallographic data for this paper.

Table S2. Crystal data and details of the structure determination for **1–3.**

**Compound**	**1**	**2**	**3**
Formula	$C_{12}H_8CdCl_2N_4$	$C_{12}H_8CdBr_2N_4$	$C_{12}H_8CdI_2N_4$
M_r	391.52	480.44	574.42
Colour and habit	colourless needle	colourless needle	colourless plate
Crystal system, space group	Monoclinic, $P2_1/c$	Monoclinic, $P2_1/c$	Monoclinic, $I2/a$
Crystal dimensions (mm^3)	0.65 x 0.02 x 0.01	0.54 x 0.14 x 0.05	0.24 x 0.14 x 0.10
a (Å)	3.7809(3)	3.8922(3)	7.9993(2)
b (Å)	15.5001(17)	7.3492(6)	13.5504(2)
c (Å)	11.6159(13)	27.145(2)	14.9321(3)
α (˚)	90	90	90
β (˚)	91.503(8)	92.365(8)	104.098(2)
γ (˚)	90	90	90
V (Å^3)	680.51(12)	775.82(11)	1569.80(6)
Z	2	2	4
D_{calc} (g cm^{-3})	1.911	2.057	2.431
μ (mm^{-1})	1.986	6.548	5.316
F(000)	380	452	1048
θ range for data collection (˚)	2.628 – 28.492	2.872 – 28.495	2.813 – 28.495
h, k, l range	−5:4, −20:20, −15:15	−5:5, −9:9, −36:36	−10:10, −18:18, −20:20
Scan type	ω	ω	ω
No. measured reflections	8958	10878	14865
No. independent reflections (R_{int})	1714 (0.0527)	1966 (0.0559)	1991 (0.0771)
No. observed reflections, $I \geq 2\sigma(I)$	1358	1591	1862
No. refined parameters	88	88	88
R, wR [$I \geq 2\sigma(I)$]	0.0250, 0.0489	0.0336, 0.0555	0.0203, 0.0522
R, wR [all data]	0.0369, 0.0511	0.0531, 0.0590	0.0220, 0.0531
Goodness of fit on F^2, S	1.029	1.019	1.068
Max., min. electron density (e Å^{-3})	0.368, −0.356	0.440, −0.352	0.774, −0.491
CCDC number	2127796	2127797	2127798

Table S3. Crystal data and details of the structure determination for **4–6.**

**Compound**	**4**	**5**	**6**
Formula	$C_{12}H_8CdCl_2N_4$	$C_{12}H_8CdBr_2N_4$	$C_{12}H_8CdI_2N_4$
M_r	391.52	480.44	574.42
Colour and habit	colourless needle	colourless needle	colourless plate
Crystal system, space group	Monoclinic, $P2_1/c$	Monoclinic, $P2_1/c$	Monoclinic, $C2/m$
Crystal dimensions (mm^3)	0.49 x 0.015 x 0.025	0.22 x 0.06 x 0.04	0.48 x 0.24 x 0.07
a (Å)	3.79490(10)	3.90130(10)	24.7082(5)
b (Å)	7.2352(2)	7.4398(2)	4.13070(10)
c (Å)	26.4504(10)	26.8454(8)	7.6033(2)
α (˚)	90	90	90
β (˚)	90.930(3)	92.374(2)	96.088(2)
γ (˚)	90	90	90
V (Å^3)	726.15(4)	778.52(4)	771.63(3)
Z	2	2	2
D_{calc} (g cm^{-3})	1.791	2.050	2.472
μ (mm^{-1})	1.861	6.526	5.407
F(000)	380	452	524
θ range for data collection (˚)	2.919– 28.493	2.738– 28.499	3.010– 28.490
h, k, l range	−5:5, −9:9, −35:35	−5:5, −9:9, −36:36	−32:32, −5:5, −10:10
Scan type	ω	ω	ω
No. measured reflections	45583	9786	14892
No. independent reflections (R_{int})	1820 (0.0559)	1795(0.0486)	1056 (0.1005)
No. observed reflections, $I \geq 2\sigma(I)$	1661	2005	1117
No. refined parameters	88	89	60
R, wR [$I \geq 2\sigma(I)$]	0.0146, 0.0342	0.0291, 0.0670	0.0246, 0.0580
R, wR [all data]	0.0173, 0.0351	0.0346, 0.0692	0.0281, 0.0604
Goodness of fit on F^2, S	1.053	1.148	1.081
Max., min. electron density (e Å^{-3})	0.258 −0.218	1.066, -0.705	0.980, −0.903
CCDC number	2127799	2127800	2127801

Table S4. Selected bond distances (Å) and angles (˚) for **1**, **2**, **4–6**.

	**1**	**2**	**4**	**5**	**6**
Cd1–N1	2.3993(19)	2.390(3)	2.3768(12)	2.389(3)	2.429(3)
Cd1–X1	2.6226(5)	2.7453(4)	2.6254(3)	2.7413(4)	2.94875(17)
Cd1–X1^{i}	2.6068(5)	2.7664(4)	2.6292(3)	2.7676(4)	2.94875(17)
N1–Cd1–X1	91.02(4)	90.68(8)	88.22(3)	91.27(8)	89.84(6)
N1–Cd1–X1^{i}	90.32(5)	89.45(8)	90.59(3)	90.41(8)	89.84(6)
N1–Cd1–X1^{ii}	89.68(5)	90.55(8)	89.41(3)	89.59(8)	90.16(6)
N1–Cd1–X1^{iii}	88.98(4)	89.32(8)	91.78(3)	88.73(8)	90.16(6)
X1–Cd1–X1^{i}	92.606(18)	89.847(12)	92.474(11)	89.827(11)	88.920(7)
X1–Cd1–X1^{ii}	87.394(18)	90.153(11)	87.526(11)	90.173(11)	91.080(7)

Symmetry codes: (*i*) *x* – 1, *y*, *z*; (*ii*) − *x* + 2, − *y* + 1, − *z* + 1; (*iii*) −*x* + 1, − *y* + 1, − *z* + 1; X = Cl (**1**)
(*i*) *x* + 1, *y*, *z*; (*ii*) − *x*, − *y* + 1, − *z* + 1; (*iii*) – *x* + 1, − *y* + 1, − *z* + 1; X = Br (**2**)
(*i*) *x* – 1, *y*, *z*; (*ii*) − *x* + 2, − *y* + 2, − *z*+1; (*iii*) − *x* + 1, − *y* + 2, − *z* + 1; X = Cl (**4**)
(*i*) − *x* +2, − *y* + 1, − *z* + 1; (*ii*) *x* − 1, *y*, *z*; (*iii*) −*x* + 1, − *y* + 1, − *z* + 1; X = Br (**5**)
(*i*) *x*, *y* − 1, *z*; (*ii*) − *x* + 1, − *y* + 1, − *z* + 1; (*iii*) – *x* + 1, −*y*, − *z* + 1; X = I (**6**)

Table S5. Selected bond distances (Å) and angles (˚) for **3**.

	**3**
Cd1–N1	2.4203(19)
Cd1–I1	2.9321(2)
Cd1–I1^{i}	3.06186(17)
N1–Cd1–I1	166.49(4)
N1–Cd1–I1^{i}	90.94(5)
N1–Cd1–I1^{ii}	89.82(5)
N1–Cd1–I1^{iii}	92.60(5)
N1–Cd1–N1^{ii}	77.78(9)
I1–Cd1–I1^{i}	84.194(5)
I1–Cd1–I1^{iii}	92.969(5)
I1^{ii} –Cd1–I1^{iii}	84.194(5)
I1^{iii} –Cd1–I1^{i}	175.456(9)
Cd1–I1–Cd1^{i}	95.805(5)

Symmetry codes: (*i*) – *x*, – *y* + 1, – *z* + 1; (*ii*) – *x* + 1/2, *y*, − *z* + 1; (*iii*) *x* + 1/2, − *y* + 1, *z*

Table S6. Details on hydrogen bond geometry (Å, °) for **1−6.**

D—H···A	d(H···A) / Å	d(D···A) / Å	∠ (D—H···A) / °	R_{HX}[a]
**1**				
C3—H3···Cl1^{i}	2.87	3.512(3)	127	0.97
**2**				
C3—H3···N2^{ii}	2.66	3.417(7)	139	0.97
C3—H3···N2^{iii}	2.81	3.447(7)	127	1.02
C5—H5···Br1^{iv}	3.13	3.781(4)	129	1.03
C4—H4···Br1^{iv}	3.22	3.813(5)	124	1.05
**3**				
C5—H5···N2^{v}	2.55	3.209(4)	128	0.93
**4**				
C4—H4···N2^{vi}	2.59	3.462(3)	156	0.94
C2—H2···Cl1^{vii}	2.91	3.5691(17)	129	0.98
C1—H1···Cl1^{vii}	3.06	3.6554(15)	124	1.04
**5**				
C2—H2···N2^{viii}	2.60	3.455(7)	153	0.95
C4—H4···Br1^{ix}	3.11	3.744(5)	127	1.02
C5—H5···Br1^{ix}	3.19	3.801(4)	125	1.04

[a] The normalized distance, R, defined according to Lommerse *et al.*[4] $R_{HX} = d(\text{H}\cdots\text{X}) / (r_H + r_X)$, where r_H and r_X are the Bondi van der Waals radii of the respective hydrogen-bond donor and acceptor atoms (H 1.20, N 1.55, Cl 1.75, Br 1.86, or I 1.98 Å) in the C—H···X hydrogen bond.
Symmetry codes: (*i*) *x*, − *y* + 3/2, *z* + 1/2; (*ii*) – *x* + 2, *y* − 1/2, − *z* + 3/2 (*iii*) – *x* + 1, *y* − 1/2, − *z* + 3/2; (*iv*) *x*, *y* − 1, z; (*v*) *x* − 1, *y*, *z*; (*vi*) – *x* + 2, *y* + 1/2, − *z* + 3/2; (*vii*) – *x* + 1, − *y* + 1, − *z* + 1; (*viii*) − *x*, *y* − 1/2, − *z* + 1; (*ix*) *x*, *y* + 1, *z*.

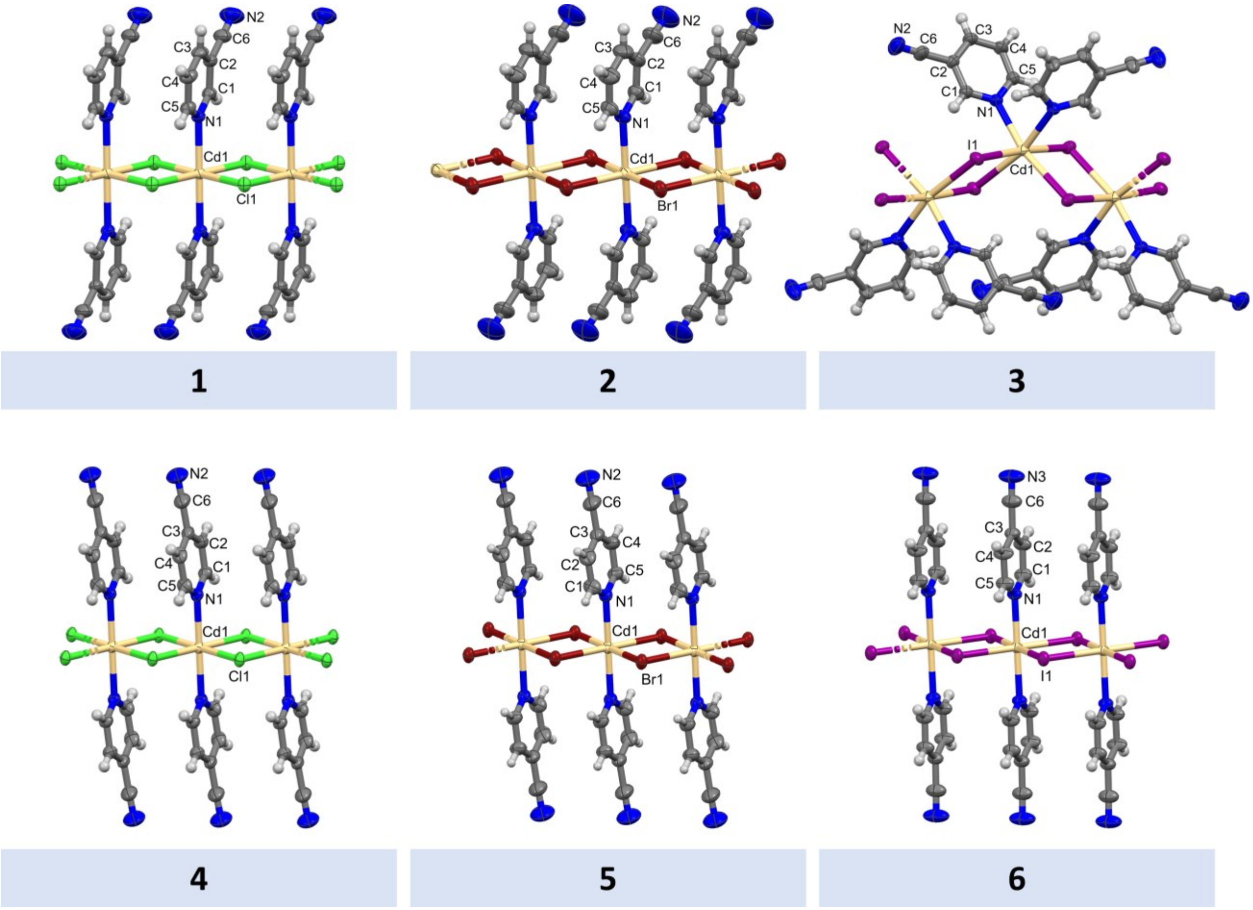

Figure S1. ORTEP-style plot of $[CdCl_2(3\text{-}CNpy)_2]_n$ (**1**) (top left), $[CdBr_2(3\text{-}CNpy)_2]_n$ (**2**) (top middle), $[CdI_2(3\text{-}CNpy)_2]_n$ (**3**) (top right), $[CdCl_2(4\text{-}CNpy)_2]_n$ (**4**) (bottom left), $[CdBr_2(4\text{-}CNpy)_2]_n$ (**5**) (bottom middle) and $[CdI_2(4\text{-}CNpy)_2]_n$ (**6**) (bottom right) with a partial labelling scheme. Thermal ellipsoids are drawn at 50% probability level at 295(2) K.

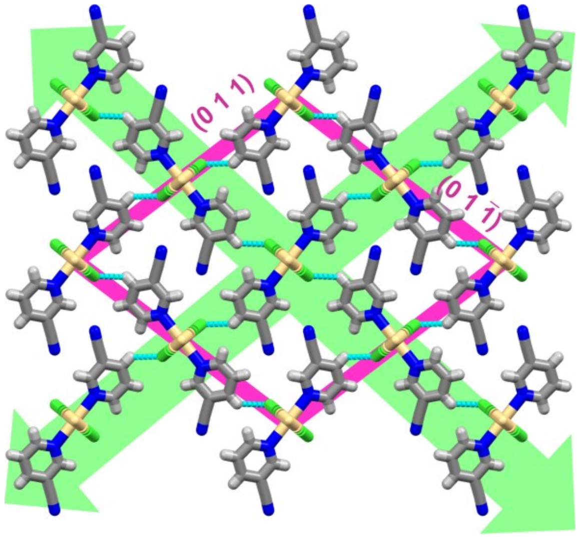

[CdCl2(3-CNpy)2]n (**1**)

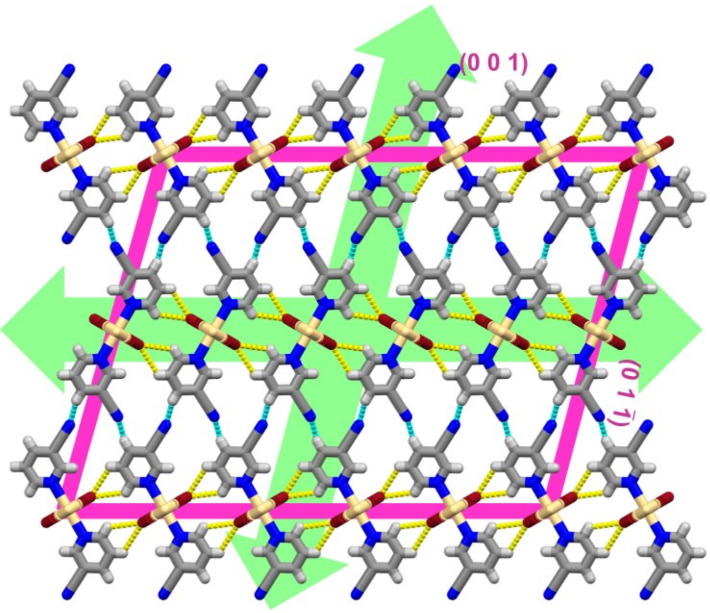

[CdBr2(3-CNpy)2]n (**2**)

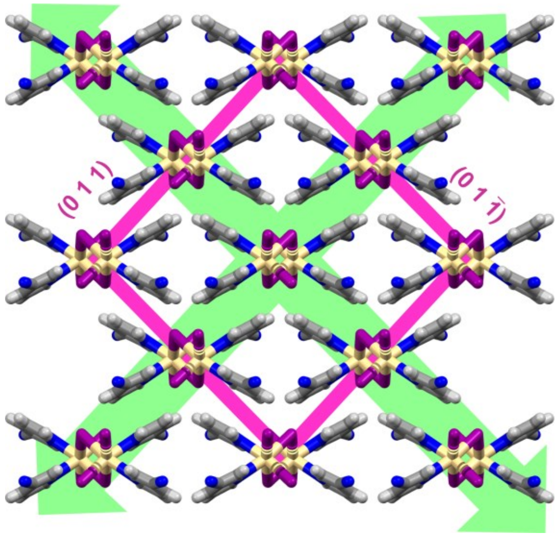

[CdI_2(3-Clpy)$_2$]$_n$ (**3**)

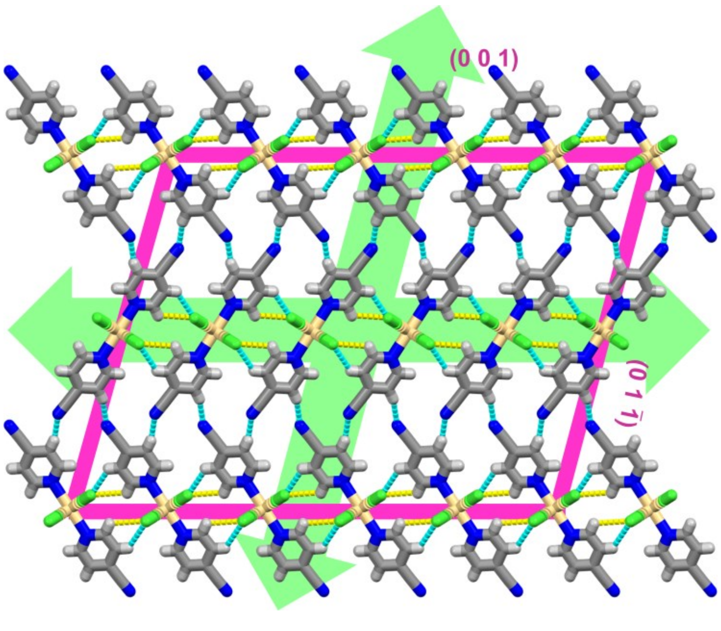

[$CdCl_2$(4-CNpy)$_2$]$_n$ (**4**)

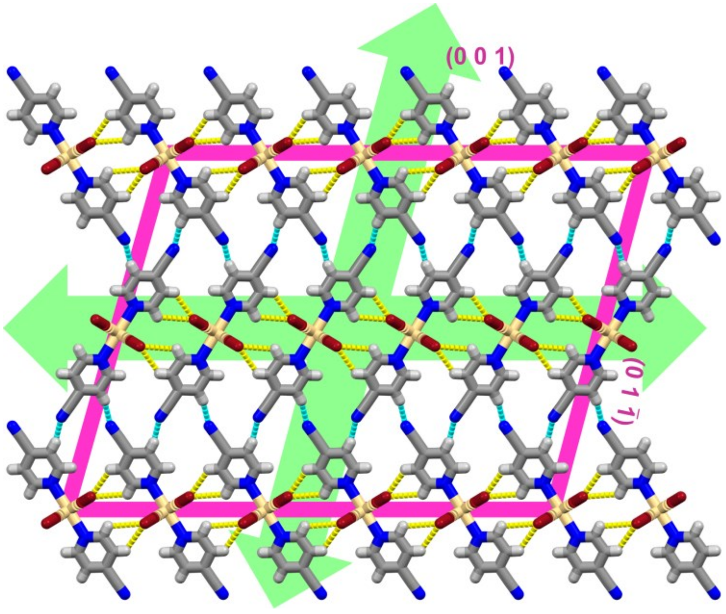

[$CdBr_2$(4-CNpy)$_2$]$_n$ (**5**)

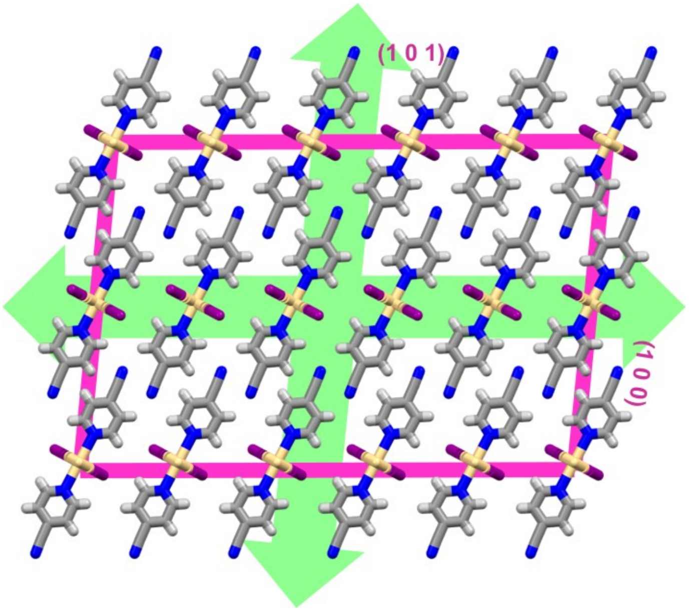

[CdI_2(4-CNpy)$_2$]$_n$ (**6**)

Figure S2. The relative orientation of adjacent polymeric chains (a view down the shortest crystallographic axis) in the crystal structures of **1**−**6** linked via hydrogen bonds forming a 2D network in the directions orthogonal to the elongation of the crystal. Intermolecular interactions that are shorter than the sum of van der Walls radii are shown as blue dotted lines, while interactions that are longer than the sum of van der Waals radii are shown in yellow.
Crystal faces are indicated by pink lines. Directions along which the mechanical force is applied are indicated by bright green arrows.

3. Powder X-ray crystallography

X-ray powder diffraction experiments were performed on a Malvern Panalytical Aeris powder diffractometer under an applied voltage of 40 kV and current of 15 mA and Cu*K*α radiation. The patterns were collected in the angle region between 5° and 50° (2θ) with a step size of 0.02°.

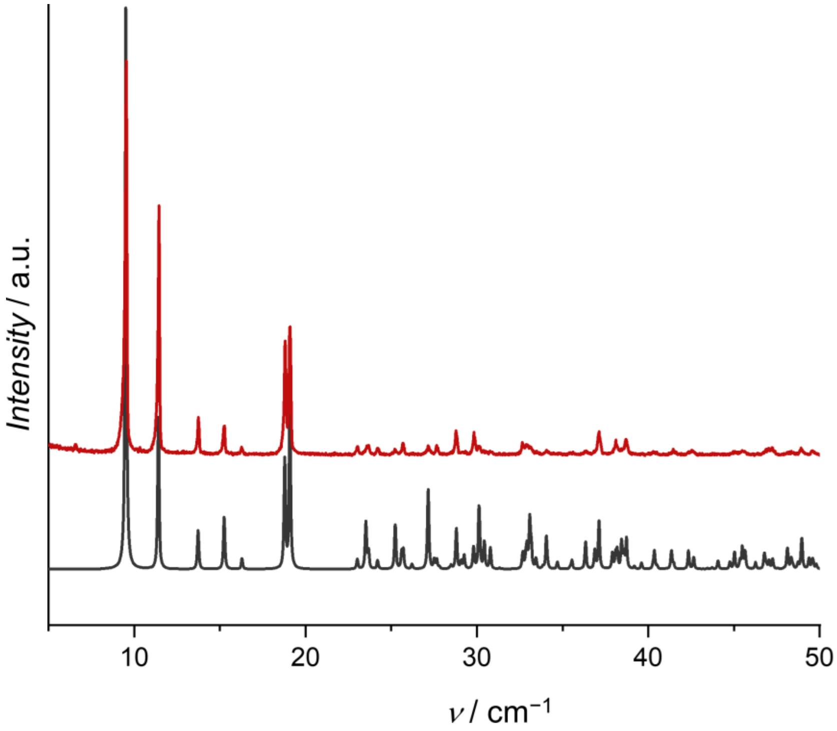

Figure S3. Experimental (**red**) and calculated (**black**) PXRD traces of $[CdCl_2(3\text{-}CNpy)_2]_n$ (**1**).

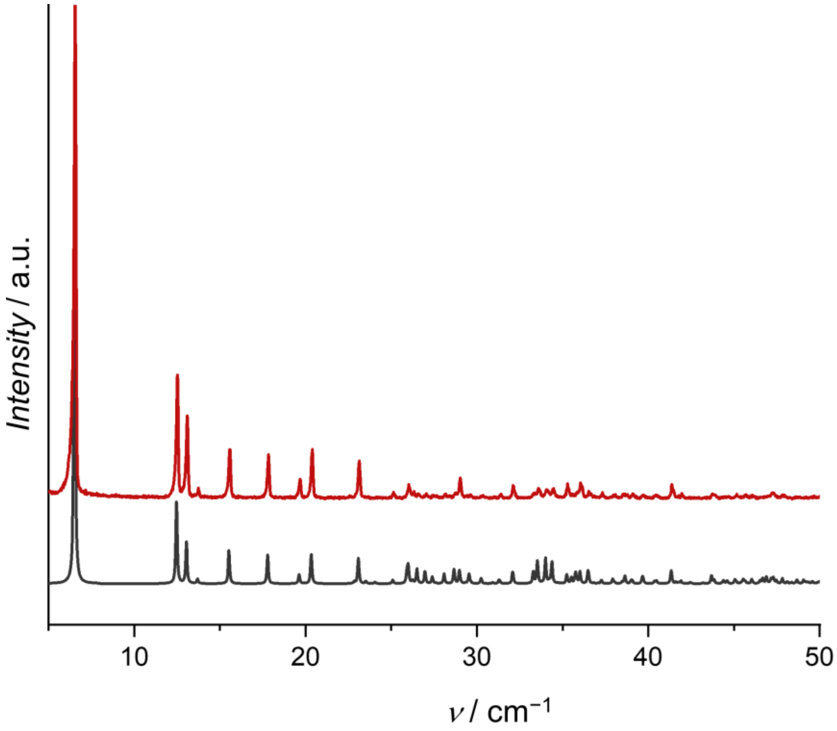

Figure S4. Experimental (**red**) and calculated (**black**) PXRD traces of $[CdBr_2(3\text{-}CNpy)_2]_n$ (**2**).

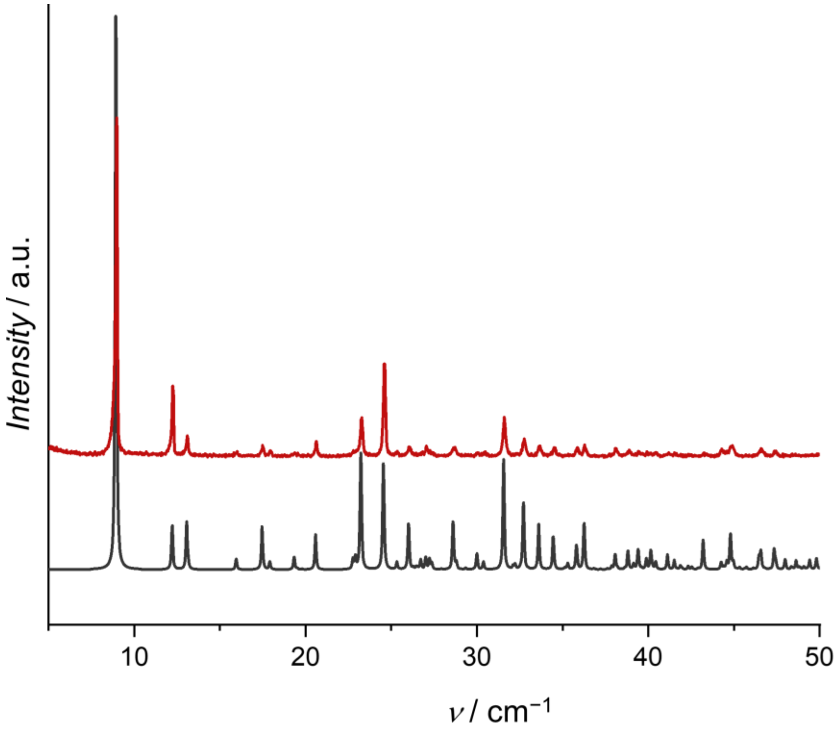

Figure S5. Experimental (**red**) and calculated (**black**) PXRD traces of $[CdI_2(3\text{-}CNpy)_2]_n$ (**3**).

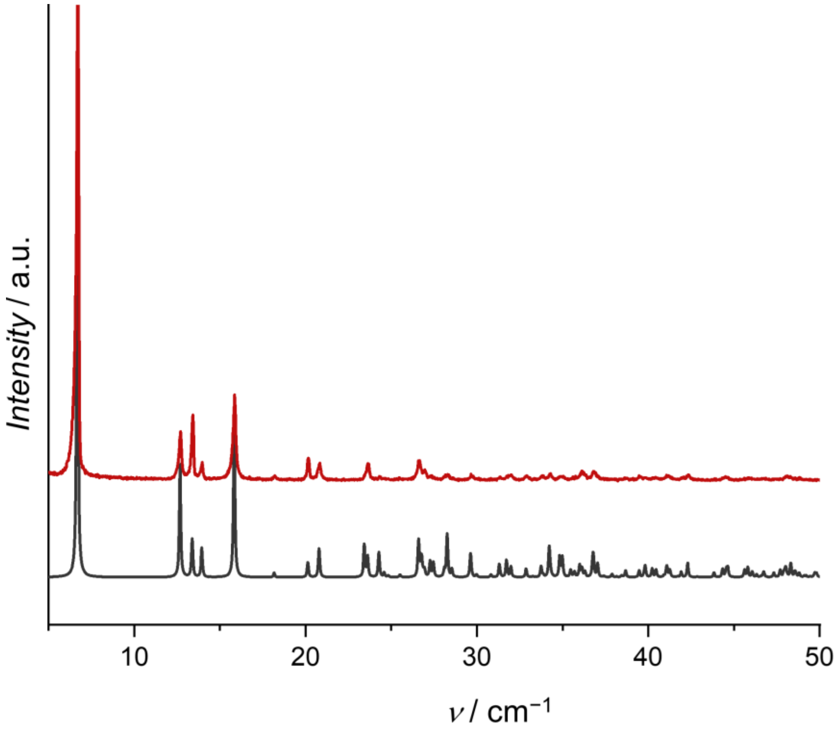

Figure S6. Experimental (**red**) and calculated (**black**) PXRD traces of $[CdCl_2(4\text{-}CNpy)_2]_n$ (**4**).

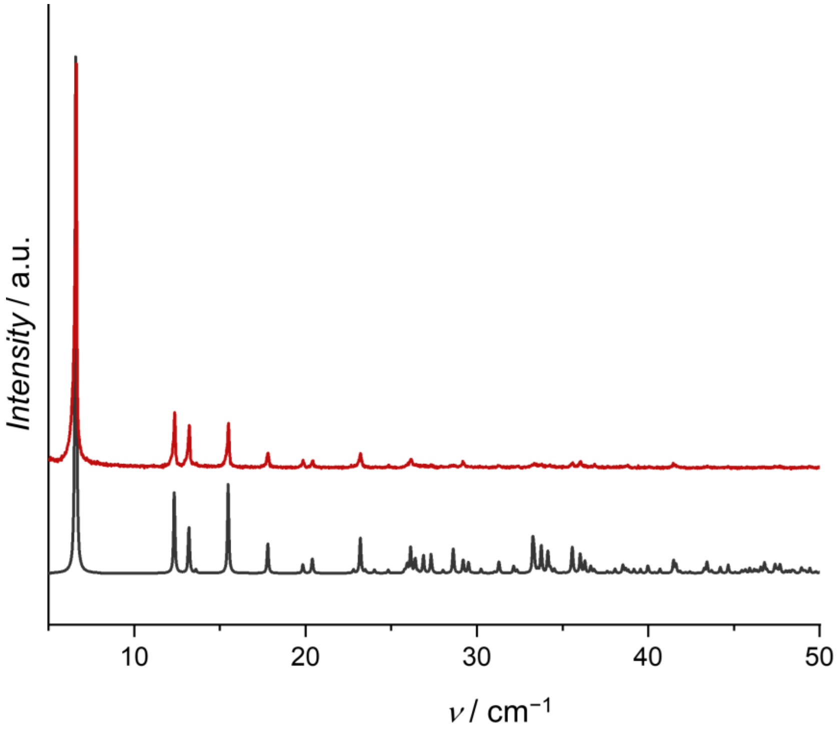

Figure S7. Experimental (**red**) and calculated (**black**) PXRD traces of $[CdBr_2(4\text{-}CNpy)_2]_n$ (**5**).

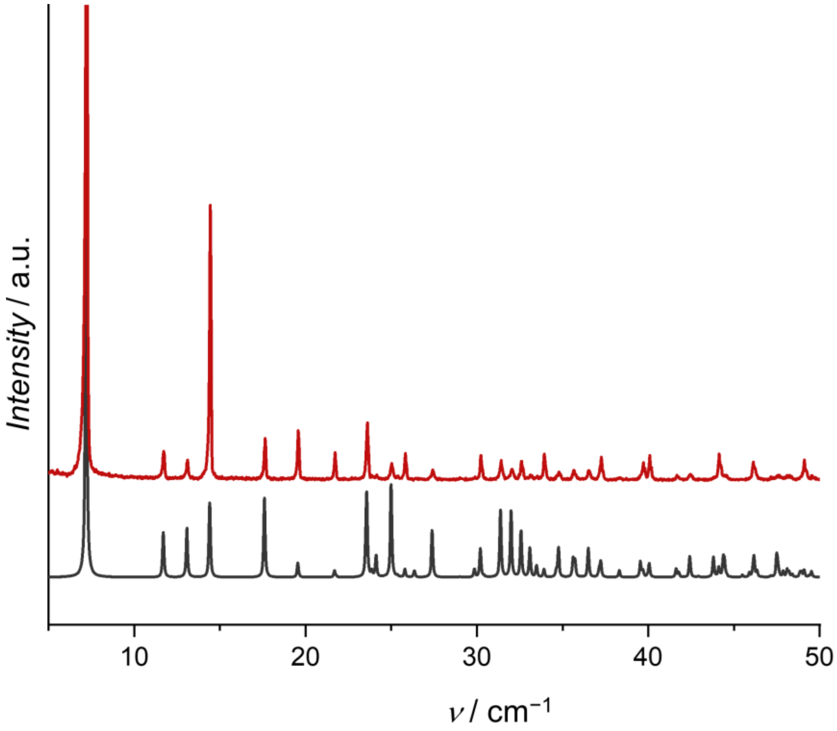

Figure S8. Experimental (**red**) and calculated (**black**) PXRD traces of $[CdI_2(4\text{-}CNpy)_2]_n$ (**6**).

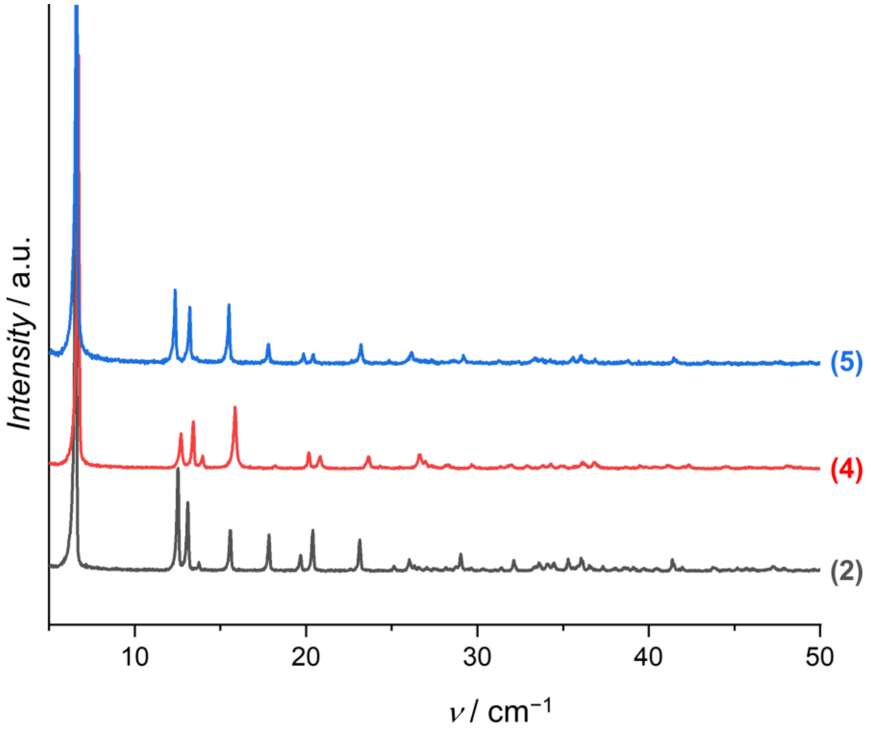

Figure S9. Comparison of PXRD traces of $[CdBr_2(3\text{-}CNpy)_2]_n$ (**2**) (**black**), $[CdCl_2(4\text{-}CNpy)_2]_n$ (**4**) (**red**) and $[CdBr_2(4\text{-}CNpy)_2]_n$ (**5**) (**blue**).

4. Thermal analysis (TGA/DTA)

Thermogravimetric analysis was performed using a simultaneous TGA-DTA analyzer Mettler-Toledo TGA/DSC 3+. Finely ground powder samples (**1−6**) were placed in alumina pans (70 μL), heated in flowing nitrogen (50 mL min^{-1}) from room temperature up to 600 °C at a rate of 10 °C min^{-1}. Data collection and analysis were performed using the program package STARe Software 16.30 MettlerToledo GmbH, 2015.

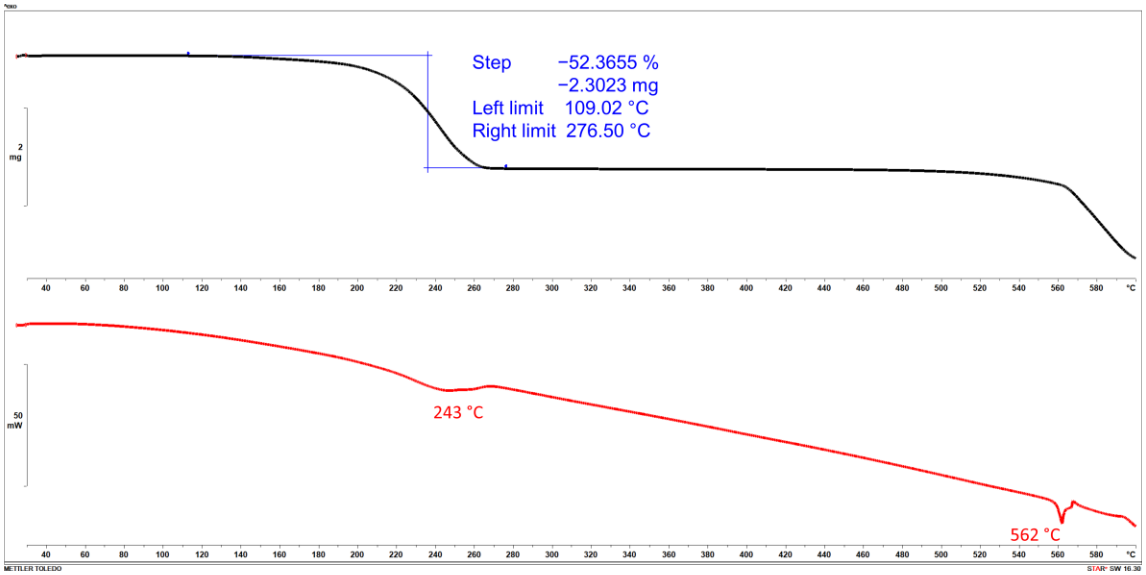

Figure S10. TGA (**black**) and DTA (**red**) curves of $[CdCl_2(3\text{-}CNpy)_2]_n$ (**1**).

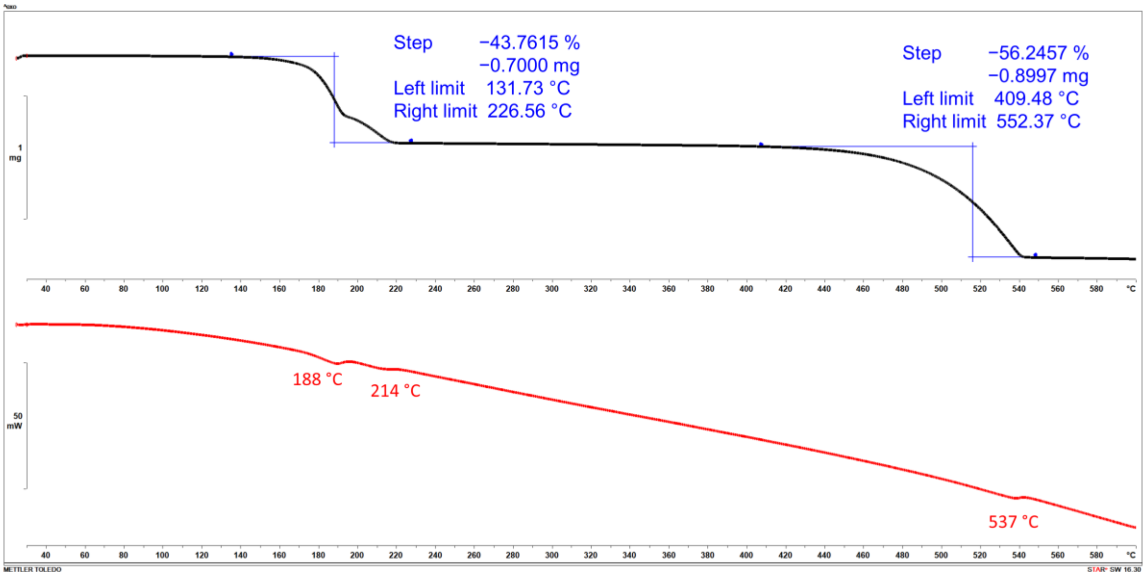

Figure S11. TGA (**black**) and DTA (**red**) curves of $[CdBr_2(3\text{-}CNpy)_2]_n$ (**2**).

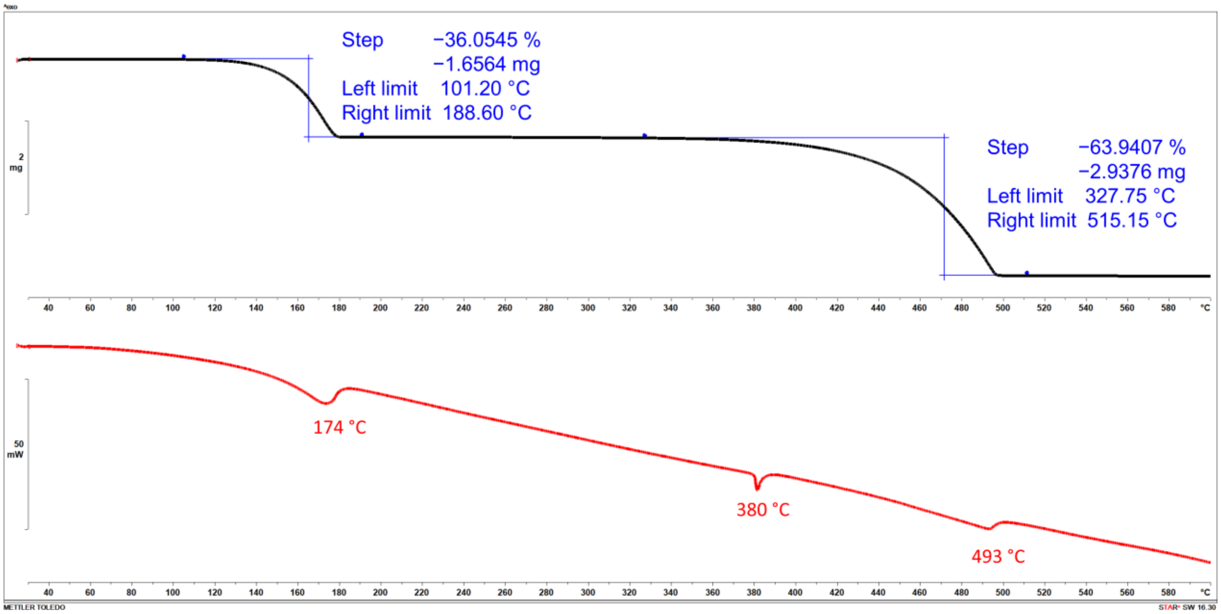

Figure S12. TGA (**black**) and DTA (**red**) curves of $[CdI_2(3\text{-}CNpy)_2]_n$ (**3**).

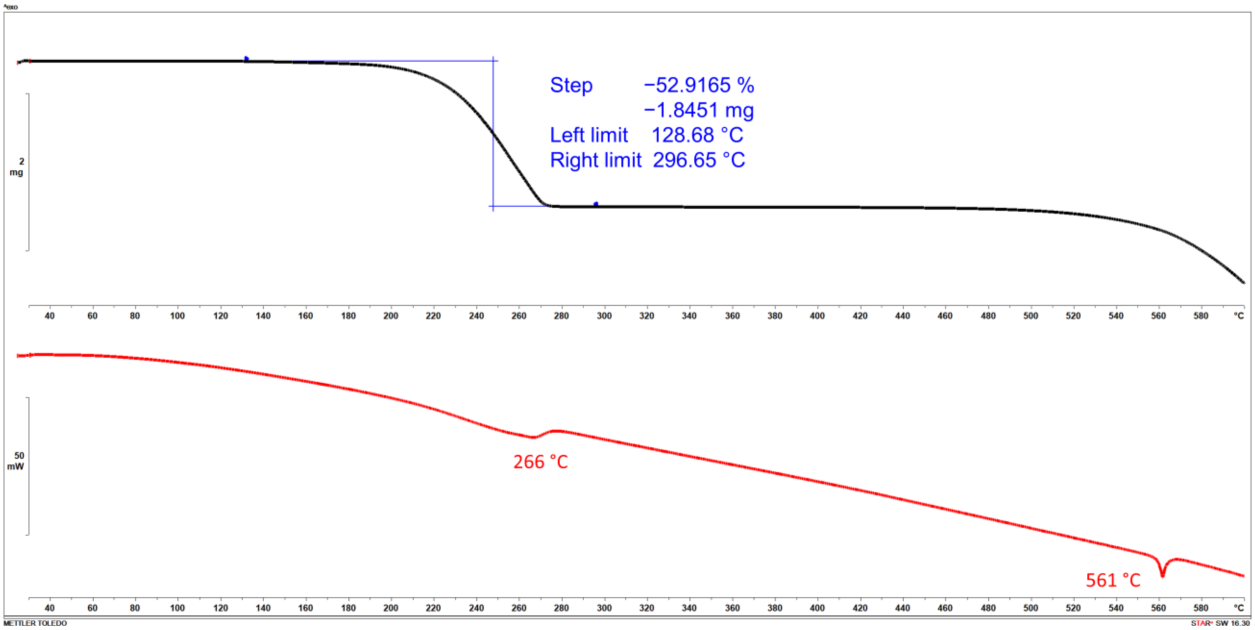

Figure S13. TGA (**black**) and DTA (**red**) curves of $[CdCl_2(4\text{-}CNpy)_2]_n$ (**4**).

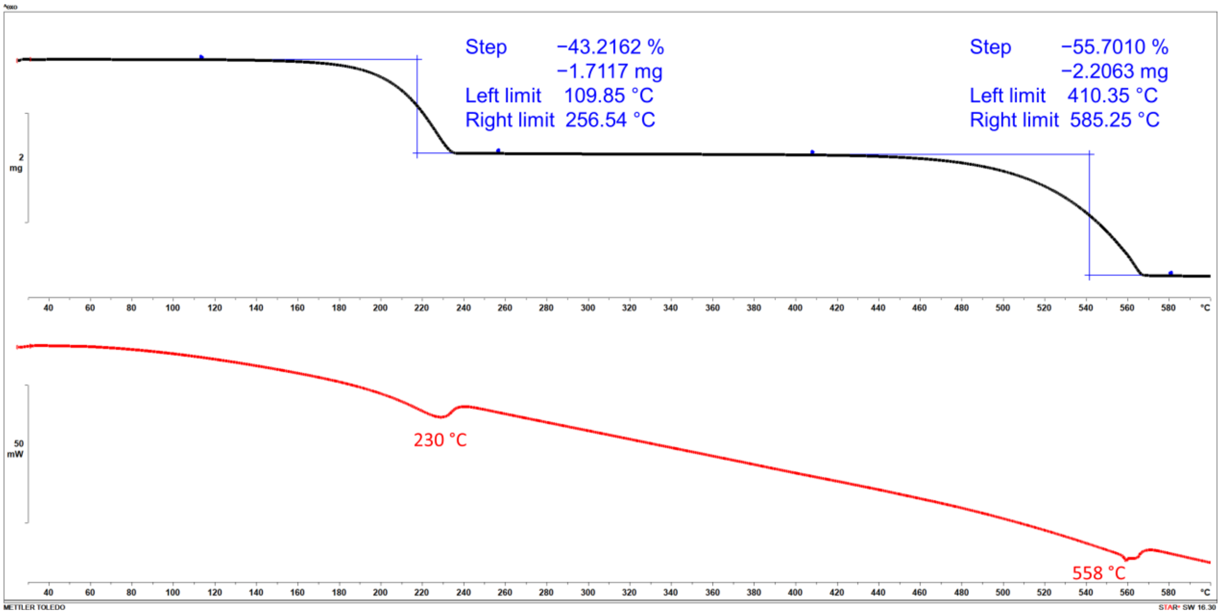

Figure S14. TGA (**black**) and DTA (**red**) curves of $[CdBr_2(4\text{-CNpy})_2]_n$ (**5**).

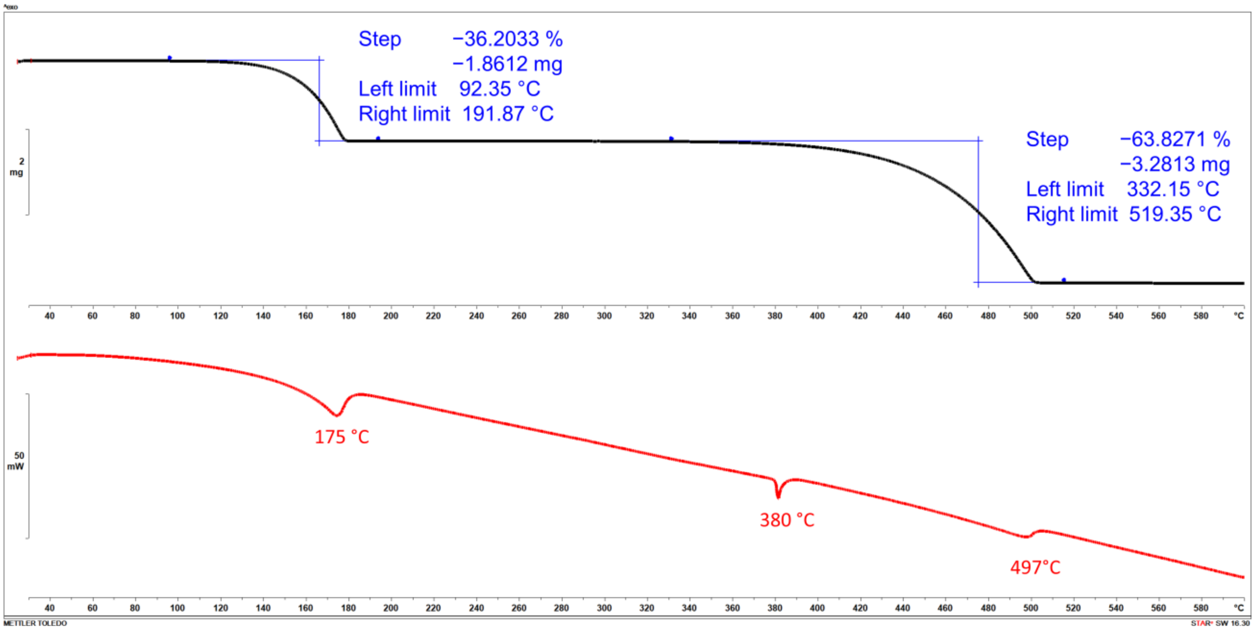

Figure S15. TGA (**black**) and DTA (**red**) curves of $[CdI_2(4\text{-CNpy})_2]_n$ (**6**).

5. Testing mechanical responses of prepared crystals

Experiments of testing crystals' responses on the application of the mechanical force were performed using a modified three-point bending procedure. Each selected crystal was placed on a glass slide and immersed in a small amount of paratone oil. From one side, the crystal was held with a pair of metal forceps, close to the crystals' ends, while from the opposite side mechanical force was applied using another pair of metal forceps with their ends held together. For crystals of each compound mechanical force was applied on both pairs of most prominent crystal faces (parallel to the elongation of the crystal), to determine the relationship between the direction of applied mechanical force and mechanical response. All bending experiments were monitored and recorded using a Dino-Lite Edge Digital Microscope (model AM4815ZT), while the recordings were taken and processed using DinoCapture 2.0 software (version 1.5.28.D).

For the crystals that showed a purely elastic response, the extent of elasticity was quantified using the Euler-Bernoulli equation.[5] The thickness (*t*) of the crystals, together with the distance between the tips of metal forceps holding the bent crystal (*L*) and maximal displacement (h_{max}) at a point of maximal curvature, i.e. just before the breakage of a crystal, were measured (Figure). The radius (*R*) of circle describing the curvature of bent crystal was calculated using the geometrical construction presented in Figure [1, 2]:

$$R^2 = \left(\frac{L}{2}\right)^2 + (R - h_{\max})^2 \qquad [1]$$

$$R = \frac{\left(\frac{L}{2}\right)^2 + h_{max}^2}{2h_{max}} \qquad [2]$$

The bending strain was calculated from Euler-Bernoulli equation [3][5] (considering pure bending without shear component):

$$\varepsilon\ (\%) = \frac{\frac{t}{2}}{R} \cdot 100 \qquad [3]$$

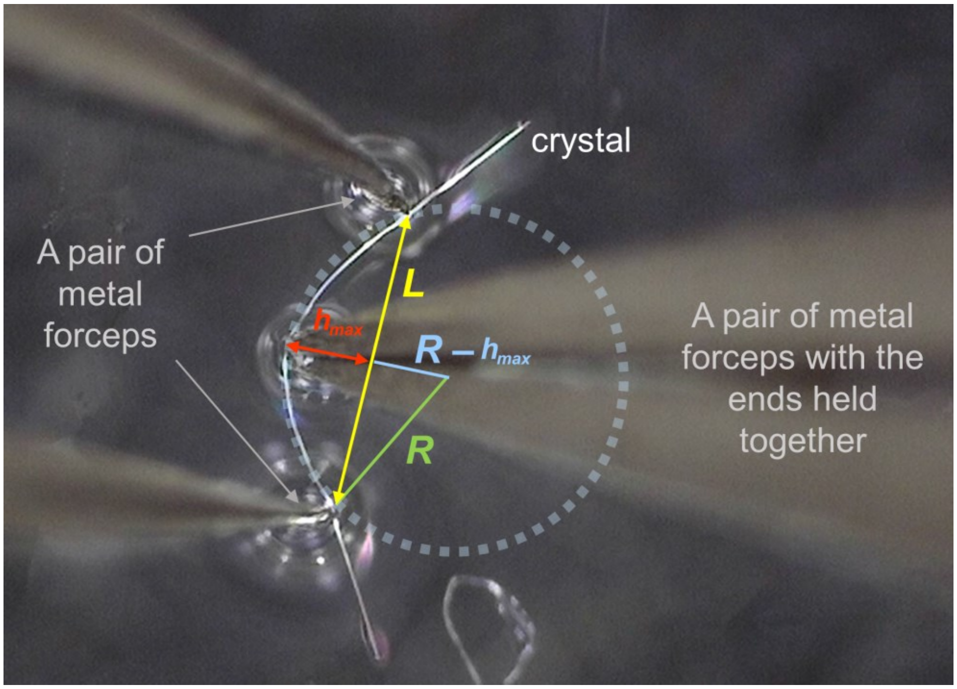

Figure S16. Three-point bending experiment highlighting measured (yellow arrow, the distance between tips of metal tweezers, *L*; red arrow, maximal displacement, h_{max}) and calculated (green line, radius of the circle (depicted as dotted gray line) approximating the curvature of the bent crystal, *R*) geometrical parameters needed for determining bending strain (ε).

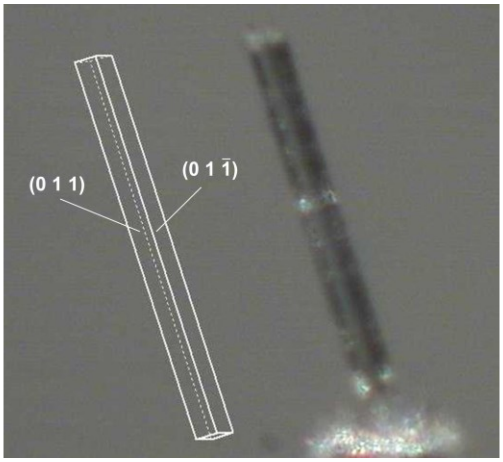

Figure S17. Face indexing for crystal of $[CdCl_2(3\text{-}CNpy)_2]_n$ (**1**).

Table S7. Geometrical parameters used to calculate the bending strain (*ε*). The mean value of the bending strain (shown in green) was determined based on measurements of five different samples for compound **1** by applying mechanical force on $(011)/(0\bar{1}\bar{1})$ or $(0\bar{1}1)/(01\bar{1})$ pair of equally developed crystal faces. The thickness (*t*) of the selected crystals was measured before bending, length (*L*) and maximal displacement (h_{max}) were measured at the point of maximal curvature (see Figure S18 below).

Sample	$\bar{t}$ / mm	*L* / mm	h_{max} /	*R* / mm	ε / %
**1-1**	0.027	1.513	0.279	1.165	**1.144**
**1-2**	0.023	1.514	0.297	1.113	**1.033**
**1-3**	0.015	1.409	0.564	0.722	**1.062**
**1-4**	0.042	1.066	0.088	1.658	**1.256**
**1-5**	0.031	1.624	0.221	1.602	**0.967**
				average	**1.093 ± 0.110**

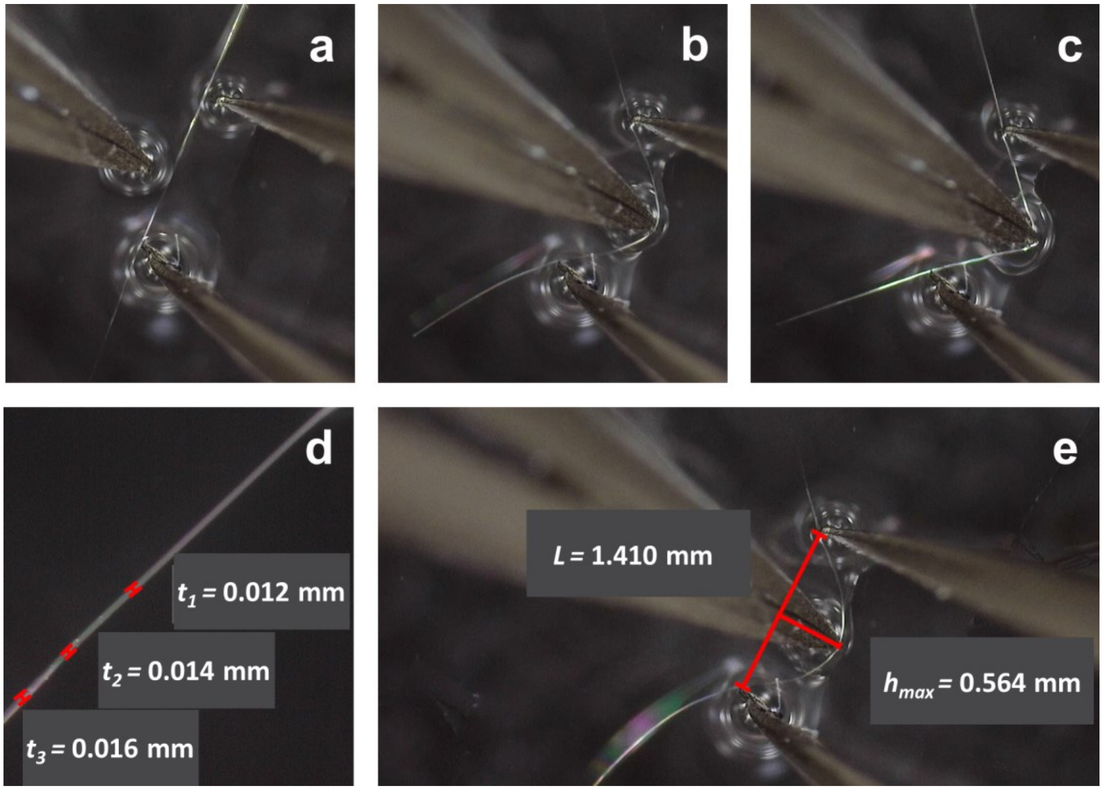

Figure S18. Bending experiment with $[CdCl_2(3\text{-}CNpy)_2]_n$ (**1**, sample **1-3**; ε = 1.06 %; images a–c, e magnified 50 times; image d magnified 200 times), by applying mechanical force on (011)/(0$\bar{1}\bar{1}$) or (01$\bar{1}$)/(0$\bar{1}$1) pair of equally developed crystal faces. Crystal bends during the application of the mechanical force till the point of the maximal curvature (a–b) while with the further application of mechanical force crystal breaks (c). At a point of maximal curvature geometrical parameters (L and h_{max}) for calculating bending strain were measured (e), while the thickness of the crystal was measured after the breakage of the crystal (d).

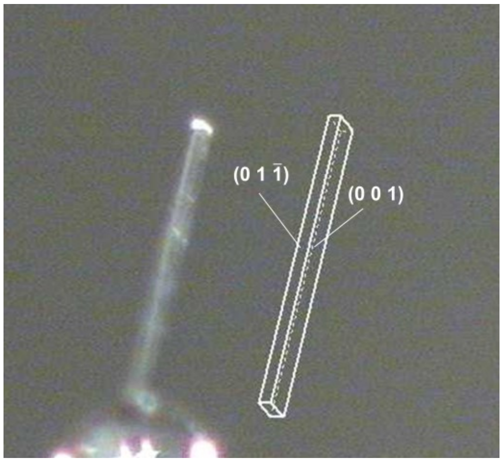

Figure S19. Face indexing for crystal of $[CdBr_2(3\text{-CNpy})_2]_n$ (**2**).

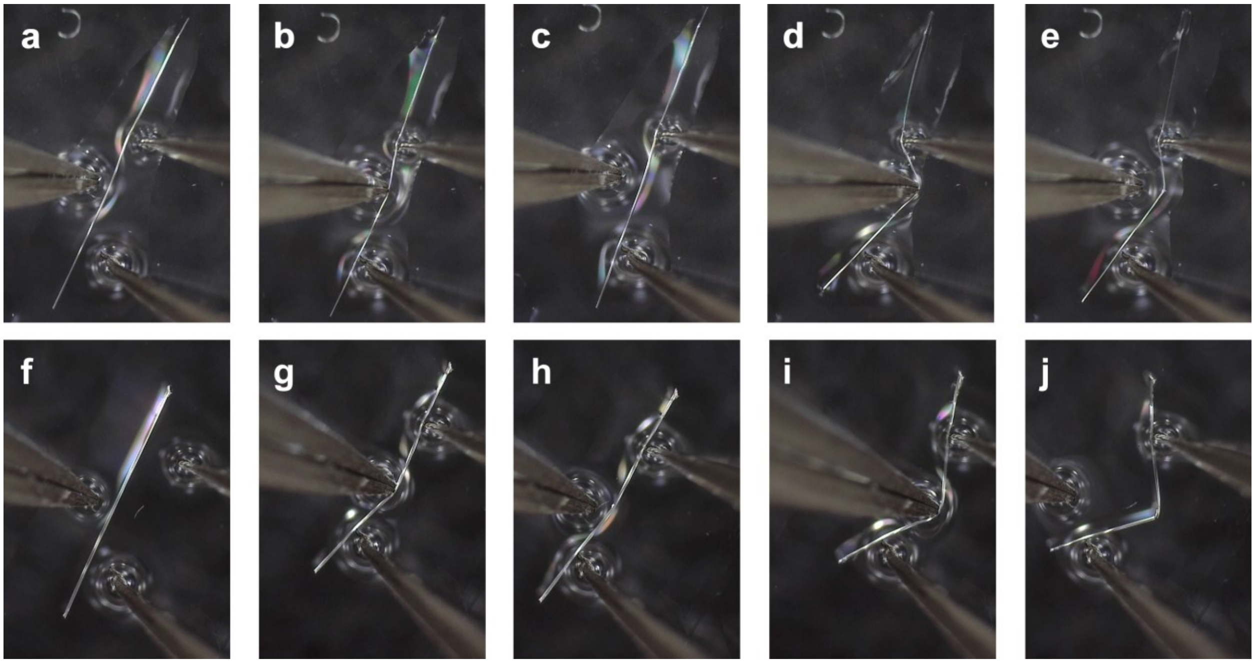

Figure S20. Bending experiment with $[CdBr_2(3\text{-CNpy})_2]_n$ (**2**) (images magnified 50 times), by applying mechanical force on $(001)/(00\bar{1})$ pair of crystal faces (a–e), and on $(01\bar{1})/(0\bar{1}1)$ pair of crystal faces (f–j). Crystal bends plastically over both pairs of differently developed crystal faces once mechanical force is applied.

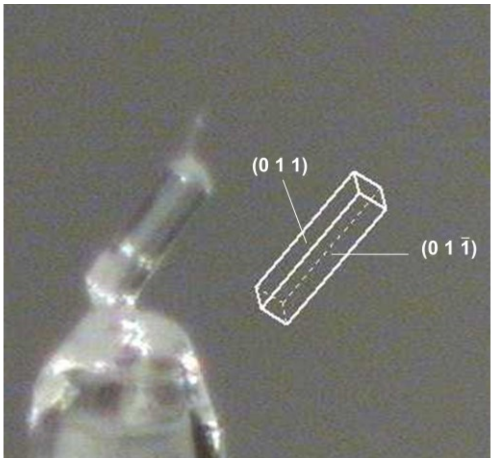

Figure S21. Face indexing for crystal of $[CdI_2(3\text{-}CNpy)_2]_n$ (**3**).

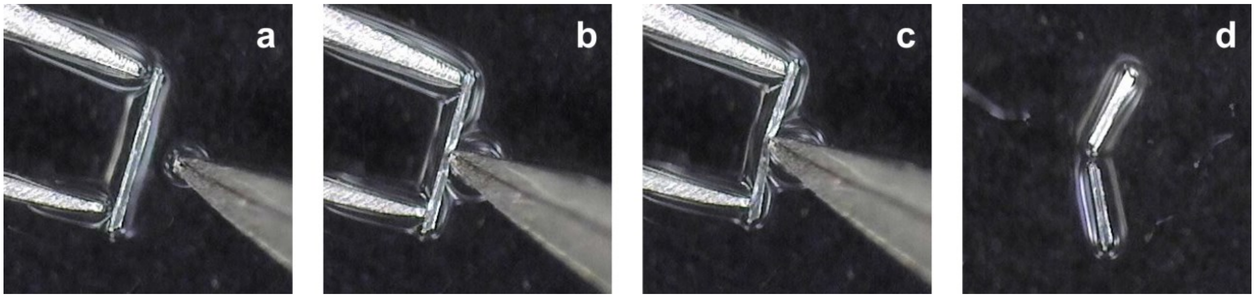

Figure S22. Bending experiment with $[CdI_2(3\text{-}CNpy)_2]_n$ (**3**) (images magnified 50 times), by applying mechanical force on on (011)/(0$\bar{1}\bar{1}$) or (0$\bar{1}$1)/(01$\bar{1}$) pair of equally developed crystal faces. Crystal breaks once the mechanical force is applied.

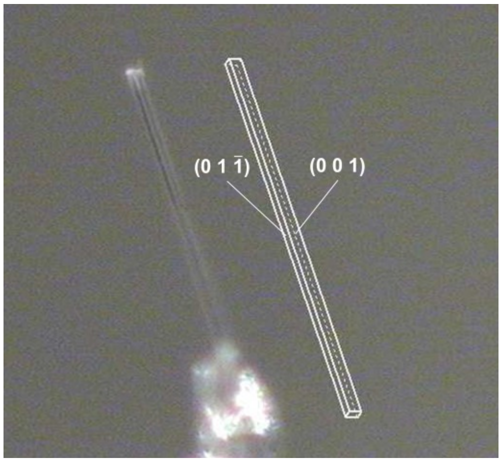

Figure S23. Face indexing for crystal of $[CdCl_2(4\text{-}CNpy)_2]_n$ (**4**).

Table S8. Geometrical parameters used to calculate the bending strain (ε). The mean values of the bending strain (shown in green) were determined based on representative measurements on ten different samples of **4**, five for bending crystals by applying mechanical force on (001)/(00$\bar{1}$) (samples **4-1** – **4-5**; ε_1), and five on (0$\bar{1}$1)/(01$\bar{1}$) pair of crystal faces (samples **4-6** – **4-10**; ε_2). Thickness (t) of the selected crystals was measured after breakage of the crystal, length (L) and maximal displacement (h_{max}) were measured at the point of maximal curvature (see Figures S24 and S25 below).

**Sample**	**$\bar{t}$ / mm**	**L / mm**	**h_{max} /**	**R / mm**	**ε / %**
**4-1**	0.015	1.354	0.381	0.792	**0.947**
**4-2**	0.030	1.459	0.220	1.319	**1.149**
**4-3**	0.031	1.468	0.257	1.177	**1.331**
**4-4**	0.016	1.477	0.480	0.808	**0.969**
**4-5**	0.012	1.239	0.626	0.620	**0.968**
				**ε_1**	**1.073 ± 0.166**
**4-6**	0.019	1.417	0.147	1.781	0.543
**4-7**	0.032	1.471	0.106	2.605	0.614
**4-8**	0.032	2.14	0.146	3.994	0.405
**4-9**	0.032	2.085	0.190	2.955	0.547
**4-10**	0.030	2.151	0.210	2.859	0.519
				**ε_2**	**0.526 ± 0.076**

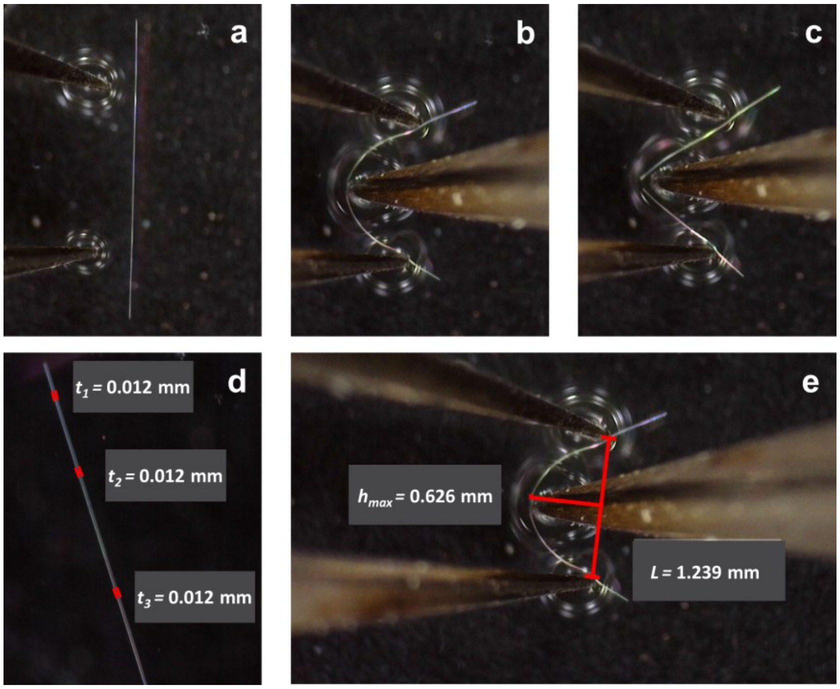

Figure S24. Bending experiment with $[CdCl_2(4\text{-}CNpy)_2]_n$ (**4**, sample **4-5**; ε = 0.97 %; images a–c, e magnified 50 times; image d magnified 200 times), by applying mechanical force on (001)/(00$\bar{1}$) pair of crystal faces. Crystal bends during the application of the mechanical force till the point of its maximal curvature (a–b) while it breaks with the further application of mechanical force (c). At a point of maximal curvature geometrical parameters (L and h_{max}) for calculating bending strain were measured (e), while the thickness of the crystal was measured after the breakage of the crystal (d).

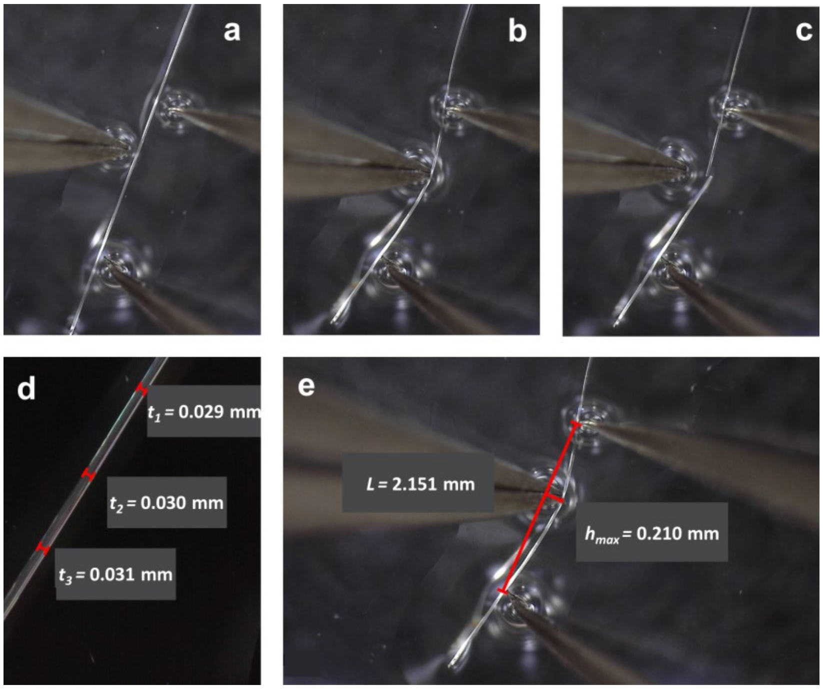

Figure S25. Bending experiment with $[CdCl_2(4\text{-}CNpy)_2]_n$ (**4**, sample **4-10**; ε = 0.97 %; images a–c, e magnified 50 times; image d magnified 200 times), by applying mechanical force on $(0\bar{1}1)/(01\bar{1})$ pair of crystal faces (d–f). Crystal bends during the application of the mechanical force till the point of its maximal curvature (a–b) while it breaks with the further application of mechanical force (c). At a point of maximal curvature geometrical parameters (L and h_{max}) for calculating bending strain were measured (e), while the thickness of the crystal was measured after the breakage of the crystal (d).

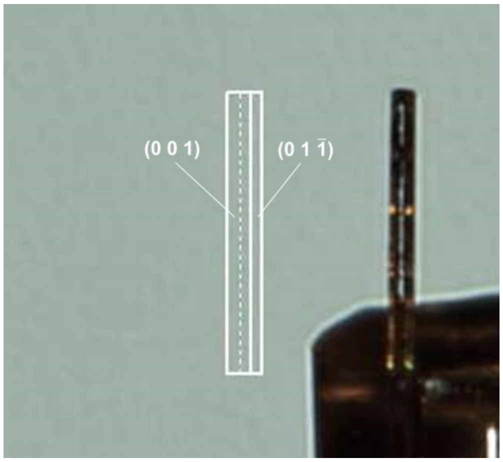

Figure S26. Face indexing for $[CdBr_2(4\text{-}CNpy)_2]_n$ (**5**).

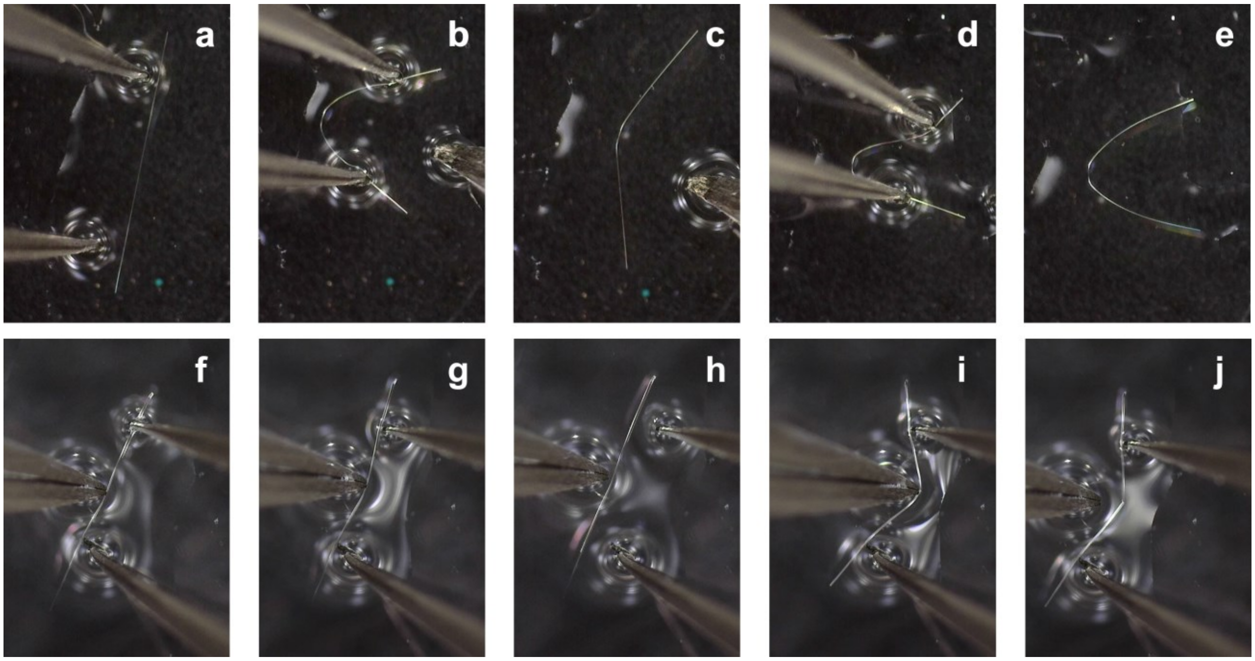

Figure S27. Bending experiment with $[CdBr_2(4\text{-}CNpy)_2]_n$ (**5**, images magnified 50 times). Plastic bending of the crystal observed while mechanical force is applied on the (001)/(00$\bar{1}$) (a–e) and (0$\bar{1}$1)/(01$\bar{1}$) pair of crystal faces (f–j).

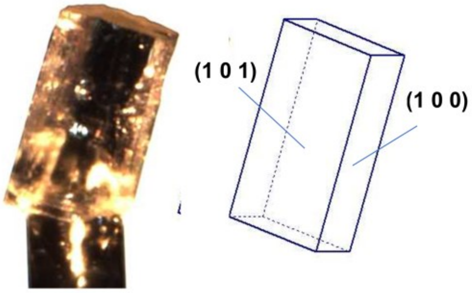

Figure S28. Face indexing for $[CdI_2(4\text{-}CNpy)_2]_n$ (**6**).

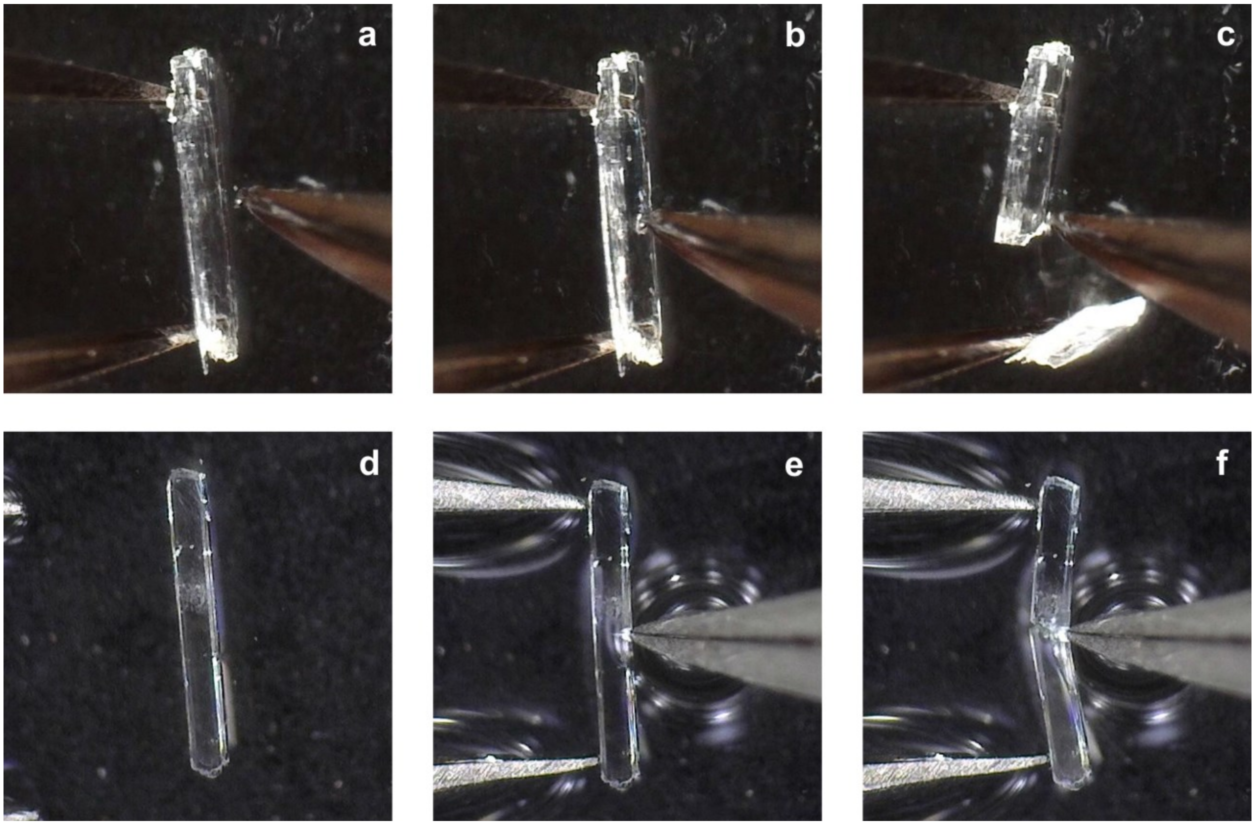

Figure S29. Bending experiment with $[CdI_2(4\text{-}CNpy)_2]_n$ (**6**), images magnified 50 times). Crystal breaks upon application of mechanical force on (101)/($\bar{1}0\bar{1}$) pair of crystal faces (a–c), and on (100)/($\bar{1}00$) pair of crystal faces (d–f).

6. Computational studies

$[CdBr_2(3\text{-}CNpy)_2]_n$ (**2**) $[CdCl_2(4\text{-}CNpy)_2]_n$ (**4**) $[CdBr_2(4\text{-}CNpy)_2]_n$ (**5**)

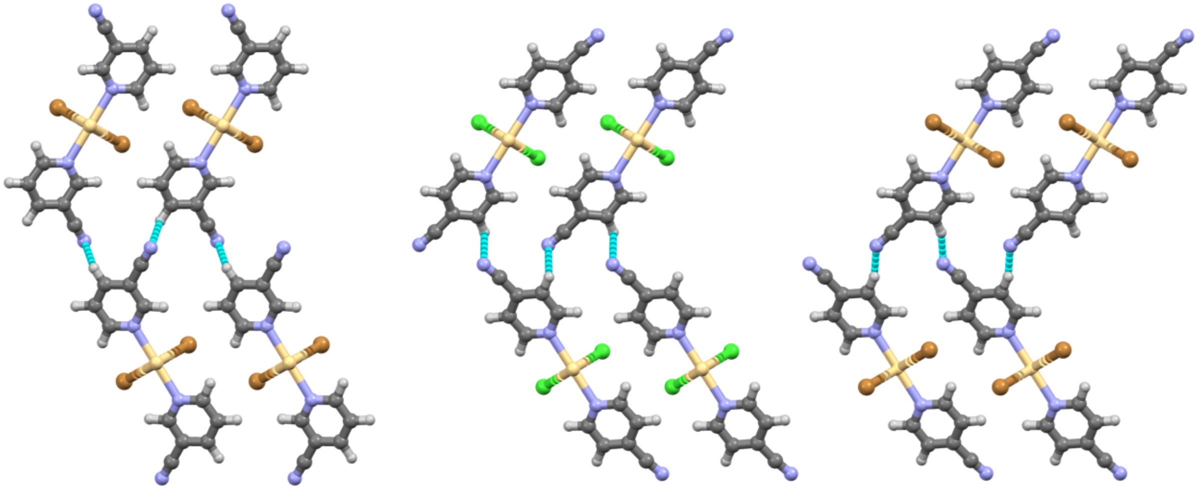

3x C−H···N(C) 3x C−H···N(C) 3x C−H···N(C)

$E_{int} = -57.5$ kJ mol^{-1} $E_{int} = -49.7$ kJ mol^{-1} $E_{int} = -51.8$ kJ mol^{-1}

Figure S30. Intermolecular interactions **A** between double pairs of truncated 1D coordination polymers in compounds **2**, **4**, and **5**. Double pairs were extracted from PBE-D3/pob-TZVP-rev2 optimized geometries in CRYSTAL17.

$[CdBr_2(3\text{-}CNpy)_2]_n$ (**2**) $[CdCl_2(4\text{-}CNpy)_2]_n$ (**4**) $[CdBr_2(4\text{-}CNpy)_2]_n$ (**5**)

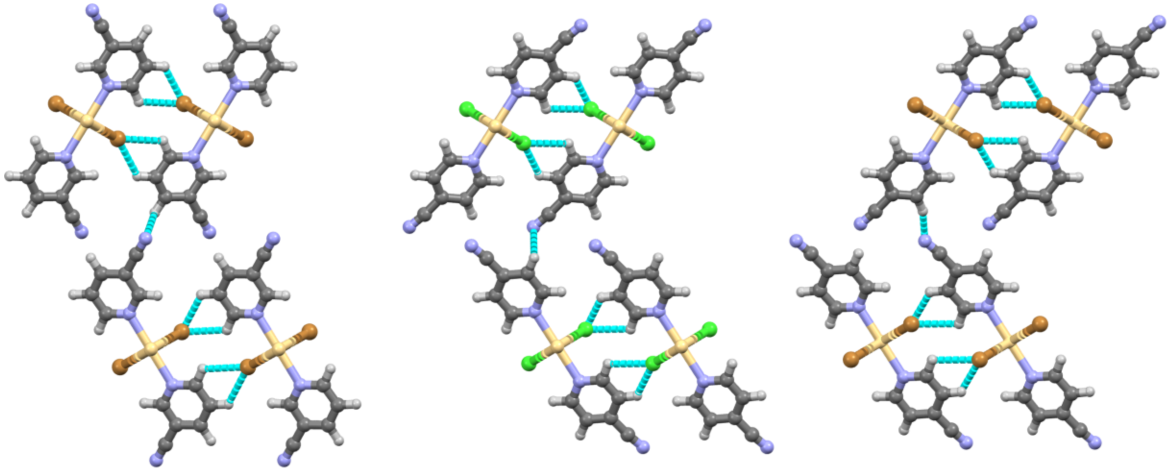

1x C−H···N(C)
8x C−H···Br(Cd)
$E_{int} = -122.7$ kJ mol^{-1}

1x C−H···N(C)
8x C−H···Cl(Cd)
$E_{int} = -160.0$ kJ mol^{-1}

1x C−H···N(C)
8x C−H···Br(Cd)
$E_{int} = -147.7$ kJ mol^{-1}

Figure S31. Intermolecular interactions **B** between double pairs of truncated 1D coordination polymers in compounds **2**, **4**, and **5**. Double pairs were extracted from PBE-D3/pob-TZVP-rev2 optimized geometries in CRYSTAL17.

TABLE S9. Fitting data associated with the Morse potential function $D_e(1 - e^{-a(X-R_e)})$ as shown in Figure 5.

compound	axis	D_e	R_e	a	k
**2**	*a*	505.455 ± 8.935	0.000 ± 0.000	0.833 ± 0.015	701.459
	b	222.121 ± 4.391	0.000 ± 0.000	0.511 ± 0.004	116.001
	c	257.948 ± 31.879	0.000 ± 0.000	0.133 ± 0.006	9.126
**4**	*a*	543.381 ± 9.190	0.000 ± 0.000	0.834 ± 0.010	755.904
	b	288.828 ± 2.054	0.000 ± 0.000	0.485 ± 0.002	135.879
	c	216.033 ± 47.490	0.000 ± 0.000	0.188 ± 0.012	15.271
**5**	*a*	492.700 ± 9.036	0.000 ± 0.000	0.839 ± 0.022	693.644
	b	259.780 ± 1.589	0.000 ± 0.000	0.472 ± 0.001	115.750
	c	223.625 ± 30.700	0.000 ± 0.000	0.173 ± 0.007	13.386

7. References

[1] Sheldrick, G. M. SHELXT– Integrated space-group and crystal-structure determination. *Acta Crystallogr*. **2015**, A71, 3.

[2] Sheldrick, G. M. Short History of ShelX. *Acta Crystallogr.,* **2008** , A64, 112.

[3] Macrae, C. F.; Bruno, I. J.; Chisholm, J. A.; Edgington, P. R.; McCabe, P.; Pidcock, E.; Rodriguez-Monge, L.; Taylor, R.; van de Streek, J.; Wood, P. A. Mercury CSD 2.0 – new features for the visualization and investigation of crystal structures. *J. Appl. Crystallogr*. **2008**, 41, 466.

[4] Lommerse, J. P. M.; Stone, A. J.; Taylor, R.; Allen, F. H. The Nature and Geometry of Intermolecular Interactions between Halogens and Oxygen or Nitrogen. *J. Am. Chem. Soc.*, **1996**, 118, 3108.

[5] Timoshenko, S. Strength of materials, D. Van Nostrand Company, New York, 1940.
